# Supplementary material for: The recombination efficiency of the bacterial integron depends on the mechanical stability of the synaptic complex
Source: Sci Adv. 2024 Dec 13;10(50):eadp8756. doi: 10.1126/sciadv.adp8756 (PMC11641012; doi:10.1126/sciadv.adp8756)
Supplement: Supplementary file 1 — Supplementary Text Figs. S1 to S14 Tables S1 to S5 Supplementary Protocols References [file sciadv.adp8756_sm.pdf]

Science Advances
AAAS

Supplementary Materials for

The recombination efficiency of the bacterial integron depends on the mechanical stability of the synaptic complex

Ekaterina Vorobevskaia *et al.*

Corresponding author: Michael Schlierf, michael.schlierf@tu-dresden.de

Sci. Adv. **10**, eadp8756 (2024)
DOI: 10.1126/sciadv.adp8756

This PDF file includes:

Supplementary Text
Figs. S1 to S14
Tables S1 to S5
Supplementary Protocols
References

Supplementary Text

High affinity of IntI1 to non-specific ssDNA

Integrase binding behavior on different DNA constructs is largely unknown. There were many studies regarding specifically recognized DNA substrates, such as *attI* sites or *attC* sites as well as how the sequence-structure interplay affects integrase binding *(10, 20, 23, 53, 54)*. However, what affinity does integrase have to the unspecific sequence, does it recognize nucleotide motifs of the *attI* or does it unspecifically bind the double-stranded DNA and perform diffusion/sliding on the molecule to bind the *attI* is unknown. We aimed to gain some initial understanding of the mode of integrase recognition and binding to an unspecific DNA substrate. We used a correlative optical tweezers setup (C-trap, LUMICKS) that has a confocal microscope to be used simultaneously with optical trapping. A DNA molecule, functionalized at the ends with biotins was tethered to the streptavidin-coated polystyrene beads and stretched to introduce small tension as well as to expose the sequence to the solution. We used our engineered fusion integrase protein that carries a monomeric enhanced green fluorescent protein ($IntI1_{mEGFP}$) to investigate how IntI1 binds the unspecific DNA substrate.

We first used a full λ-phage DNA sequence as the substrate. This DNA molecule is long (48.5 kbp) allowing for better imaging conditions and has several motifs that mimic the *attI* site. The *attI* binding box sequence allows variability, the consensus is six to seven nucleotides with conserved GTT triplet followed by three to four purines and one pyrimidine base (*29*). Such sequence flexibility results in a higher probability of finding such binding motifs on a long DNA molecule. In fact, on the λ-phage DNA, the GTTRRRRY motif is found 29 times. We tethered the molecule (biotinylated double-stranded DNA, LUMICKS) to the beads and stretched to 20 pN to expose the sequence to the $IntI1_{mEGFP}$ protein (c = 50 nM). However, no binding was detected. We repeated the same procedure on another DNA construct of a shorter length (20452 bp, biotinylated single-stranded DNA, LUMICKS) with biotin functionalization on the ends of the same DNA strand. This type of attachment provides the possibility to transform the dsDNA into a ssDNA while already in the sample chamber of the setup. The DNA was attached again to the streptavidin-coated beads and then overstretched to melt away one of the strands. Once melted, the molecule that remains tethered to the beads is purely single-stranded. We exposed such molecule to 50 nM $IntI1_{mEGFP}$ first in its dsDNA state – and again saw no binding. Then we overstretched the molecule to produce a ssDNA and exposed it again to the same $IntI1_{mEGFP}$ solution. The results were unexpected, as we observed multiple binging events along the whole length of the ssDNA molecule (Fig. S11).

The binding occurred in a wide range of forces, from 5 pN to 40 pN, and was quite durable (over 2.5 minutes). A bound $IntI1_{mEGFP}$ monomer has remained bound even upon the stretching of the molecule in the indicated range. We observed no detectable translocation of the protein along the DNA molecule based on recorded kymographs. We used the same DNA construct to probe how integrase would behave in the presence of the hybrid, overstretched DNA. We first exposed the dsDNA molecule to the $IntI1_{mEGFP}$ solution with a small tension of 7 pN, again we observed no

integrase binding. Then we started to overstretch the tether with small stalls at 15 and 30 pN to allow for some interaction, however, none was observed. We finally brought the tension to 65 pN and saw the beginning of DNA melting with occasional release of tension due to the appearance of local openings of the DNA strands. After a minute of the melting process, keeping the tether at ~65 pN we observed first $IntI1_{mEGFP}$ binding events. Those appeared on the tether closer to the beads, corresponding to the opening of melted DNA bubbles along the molecule. Some interactions were again very stable, $IntI1_{mEGFP}$ remained bound at 65pN DNA tension for over two minutes (Fig. S11B). Once the tether broke, we had no more binding signal from the $IntI1_{mEGFP}$ between the beads. With this preliminary data on the integrase binding behavior, we discovered that it has a surprisingly high affinity to unspecific ssDNA, independent of the tension forces acting at the molecule. On another hand, we observed, unexpectedly, no binding of $IntI1_{mEGFP}$ to the unspecific as well as *attI*-like dsDNA sequences.

Mechanical work during synaptic complex disassembly

We have characterized the stability of synaptic complex using disassembly force and found that lower efficiency *attC* sites showed at least 5 pN lower mean disassembly force. This is approximately 0.6 times lower than the reference high-efficiency $attC^{bs}_{aadA7}$. To better present the difference between the biochemical and biophysical processes that occur in the cell we aimed to assess the energetics of the transition from a synaptic complex to its disassembled state (the hairpin-bound state). We calculated the total mechanical work ($\Delta W = \Delta W_{dissipated} + \Delta W_{reversible}$) for all investigated protein-DNA combinations and presented the results in biologically relevant units of k_BT. The mechanical work provides us with insights on the energy difference between two stable states in our non-equilibrium experiments; the synaptic complex and the hairpin-bound state. As we assume the latter to have the same Gibbs free energy for all our *attC* sites, ΔW manifests into the different stabilities of the synaptic complex. With more work during the transit from the synaptic assembly to the hairpin-bound state, we infer a more energetically favorable synaptic complex assembly (a state with lower Gibbs free energy). Thus, using mean mechanical work ($\overline{\Delta W}$) we have built an energy profile with varying stabilities for synapses containing different *attC* sites or mutant variants.

All $\overline{\Delta W}$ values remain in a range of 62-124 k_BT, which suggests that there is a large amount of dissipation ($\Delta W_{dissipated}$) This is a hallmark for hysteresis which has several implications in biology out of equilibrium. As expected from the force difference, the low recombination efficiency *attC* sites did not vary much between themselves in terms of $\overline{\Delta W}$ ranging from 78 k_BT to 64 k_BT for $attC^{ts}_{aadA7}$ and $attC_{VCR2/1}$ (Fig. S13C). Interestingly, a synaptic complex containing a highly efficient $attC^{bs}_{aadA7}$ and IntI1 required 1.5 times more energy than its counterpart $attC^{ts}_{aadA7}$. However, exchanging the protein to the C-terminal deficient $IntI1^{\Delta C}$ decreased the work of the disassembly twice ($\overline{\Delta W}$ = 62 k_BT), likely increasing the Gibbs free energy of the synaptic formation and making it less favorable. Very close work values were obtained for low recombinogenic $attC_{VCRinv}$ ($\overline{\Delta W}$ = 67 k_BT), that differs four orders of magnitude from the $attC^{bs}_{aadA7}$ *in vivo*. We must note that as the C-terminal deletion can theoretically influence the hairpin-bound formation by destabilizing the

dimer assembly on the *attC*, the above comparison should be taken with caution. We cannot be certain that the hairpin-bound state has the same Gibbs free energy for the integrase mutant due to altered protein interactions. Alanine mutant $IntI1^{A}$ has mutations that are not expected to manifest in the hairpin-bound state as the αI2 helix residues that were changed are involved in the *attC* interaction *in trans* and do not play a role in the hairpin binding alone.
The mechanical work (W) was calculated as the integral of force (F) function over displacement (x) (eq. 1).

$$W = \int_{x0}^{x1} F(x)dx \qquad (1)$$

For this, an hWLC was plotted for a synaptic complex-bound state (S) and the next stable hairpin-bound state (HB). The integral was calculated for both curves separately for x in the range from 0 to the distance of the disassembly event (x_1) producing W_S and W_{HB}. The work done by the tether upon synaptic complex disassembly was calculated by subtraction of the work done by the tether from the HB state forth (W_{HB}) from the synaptic complex state work W_S. This ΔW is the synaptic complex disassembly work (Fig. S13) that depends on the F_{diss} (synaptic complex disassembly force). Every measuring condition (the combination of an *attC* and a protein variant) has its characteristic unfolding force dataset that we use to obtain the corresponding characteristic work values. It was recalculated to k_BT to make it more relevant in the context of biological processes. Free energy profiles were constructed as a model representation using synaptic complex disassembly work (ΔW) as an energy term of transition between the states (*55*). The reaction coordinate is arbitrary.

Synaptic complex stability using wildtype-like integrase

We performed all our measurements using a catalytically inactive integrase $IntI1^{Y312F}$ to avoid potential cleavage of the synaptic complex as possible on the same DNA molecule. However, as the naturally occurring synaptic complex in the cell is formed by the wildtype, catalytically active IntI1 ($IntI1^{wt}$), we investigated the corresponding synaptic complex stability. We used the double-*attC* construct with the reference $attC_{aadA7}{}^{bs}$ site in the established OT stability assay. Exposing the tether to 120 nM of $IntI1^{wt}$, lead to the formation of synaptic complexes of similar appearance as for the non-catalytic variant $IntI1^{Y312F}$. Disassembly forces were widely distributed as observed for IntI1 (Fig. S14). $\bar{F}_{\text{diss}}$ ($IntI1^{wt}$) = 15.6 pN ± 1.3 pN (N = 90, mean ± SEM), did not differ from IntI1 significantly, tested using the Welch T-test (p-value = 0.23), Student T-test (p-value = 0.18) and Kruskal-Wallis non-parametric test (to take into account non-normal distribution of the datasets, p-value = 0.97).

Double-attC construct unfolding behavior in the absence of IntI

For reliable synaptic complex disassembly measurements, it is important to establish a protein-free baseline behavior of the tether. We have performed force-spectroscopy experiments on the double-hairpin construct unfolding behavior and established that the DNA construct with two folded imperfect ssDNA *attC* hairpins can unfold in up to four different steps. In an earlier study by Mukhortava, Pöge et al. (*13*), we showed that each *attC* has a two-part unfolding pattern at low

forces: each part of the hairpin results in a 20-21 nm contour length change; a fully unfolded *attC*$_{aadA7bs}$ shows a ΔL_c ~ 41 nm (Fig. S6). Assuming two hairpin unfolding events, we would expect a contour length changes of ~ 82 nm. We collected data of 177 stretching traces that were in good agreement with the expected contour length change. In brief, the most common length change was in the range of partial unfolding (ΔL_c ~ 20-25 nm); another frequent length change was ~ 40-45 nm, originating either from a full hairpin unfolding or a nearly simultaneous partial unfolding of two hairpins. Taking into account the total contour length change, we frequently observed ~ 82 nm (Fig. S6). None of these traces showed our characteristic molecular fingerprint of ΔL_C~ 71nm, in agreement that this fingerprint is caused by the disassembly of a synaptic complex. (Fig. S6). In agreement with the earlier study (*13*), we observed low unfolding forces ranging from 2 pN to 7 pN (mostly < 6 pN), very rarely we observed unfolding events above 10 pN.

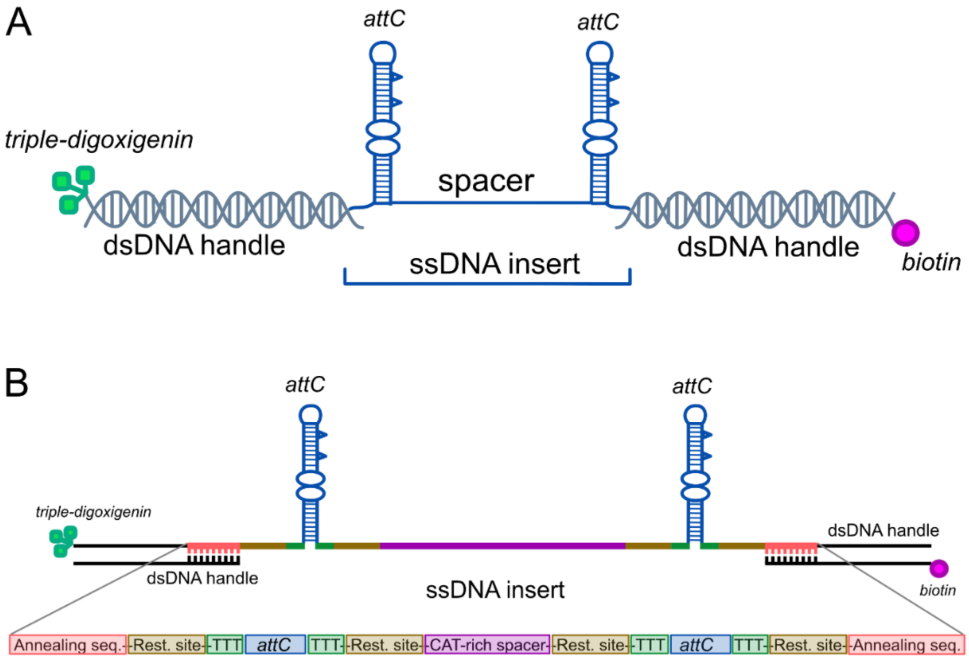

Fig. S1.

Scheme of the hybrid DNA tether assembly and composition. (A) Hybrid DNA construct for *in vitro* synapse reconstitution: two *attC* hairpins, a spacer for gene cassette imitation, dsDNA handles with 5' modifications carrying biotin and triple-digoxigenin for specific bead attachment in the optical tweezers assay. **(B)**. Schematic representation of the single-stranded insert. Two *attC* hairpins are indicated as well as the ssDNA spacer. The position of three thymine bases (TTT), restriction sites (Rest. site), the CAT-rich spacer, and complementary overhangs (Annealing seq.) are labeled and indicated with colored rectangles.

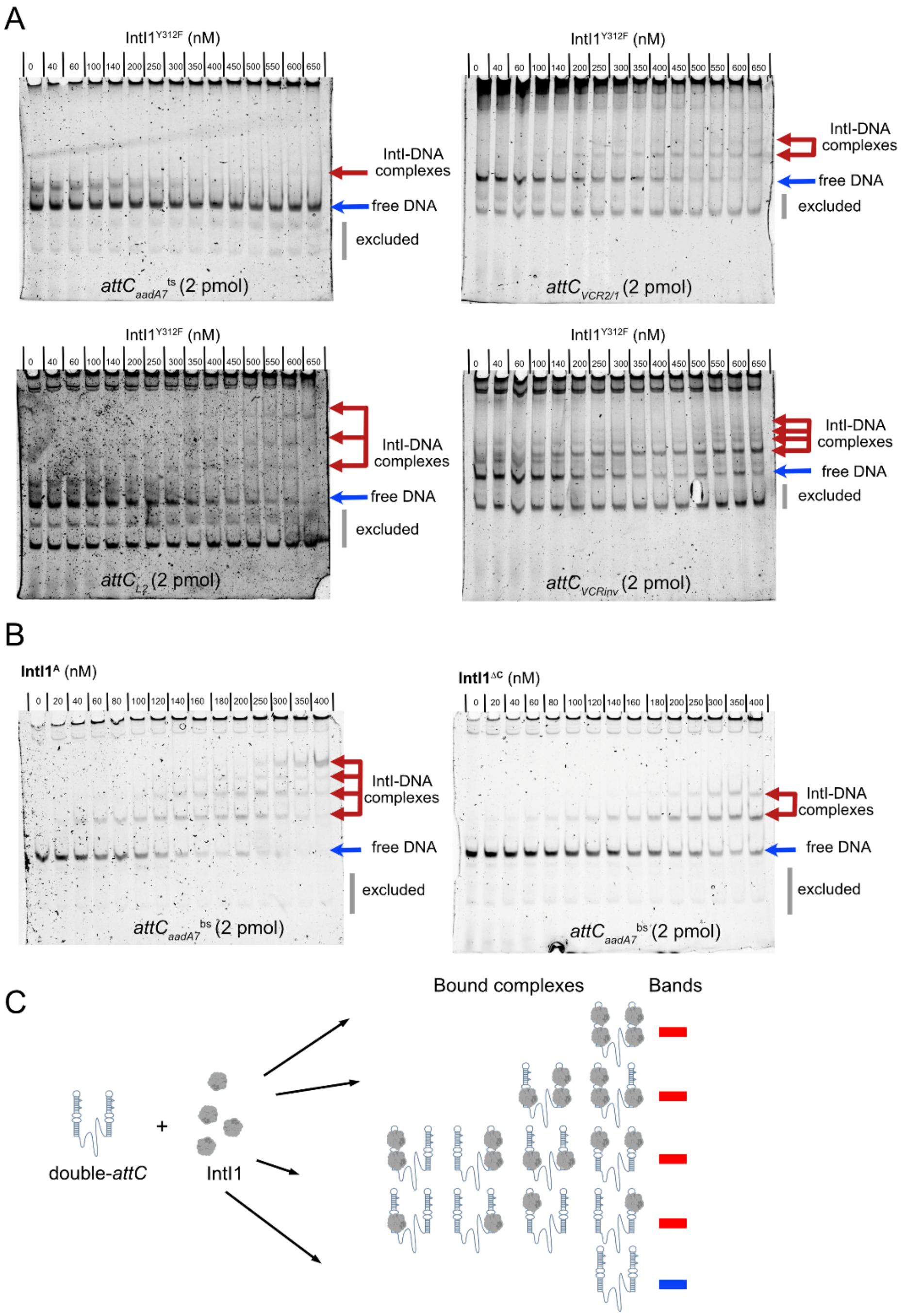

Fig. S2.

EMSA of different *attC* sites with IntI1 variants. (A) EMSA with IntI1 on $attC^{ts}_{aadA7}$, $attC_{L2}$, $attC_{VCR2/1}$, and $attC_{VCRinv}$ showed from one to four integrase subunit binding events. Bands arising from partial double-*attC* substrate are excluded from further analysis (grey). **(B)** EMSA with IntI1^{A} and IntI1$^{\Delta C}$ on $attC^{bs}_{aadA7}$ showed from one to four subunit binding modes. Bands arising from staple oligos or partial double-attC substrate are excluded from further analysis (grey) **(C)** Schematic representation of possible binding arrangements of IntI1 to double-*attC* DNA construct including molecular heterogeneity.

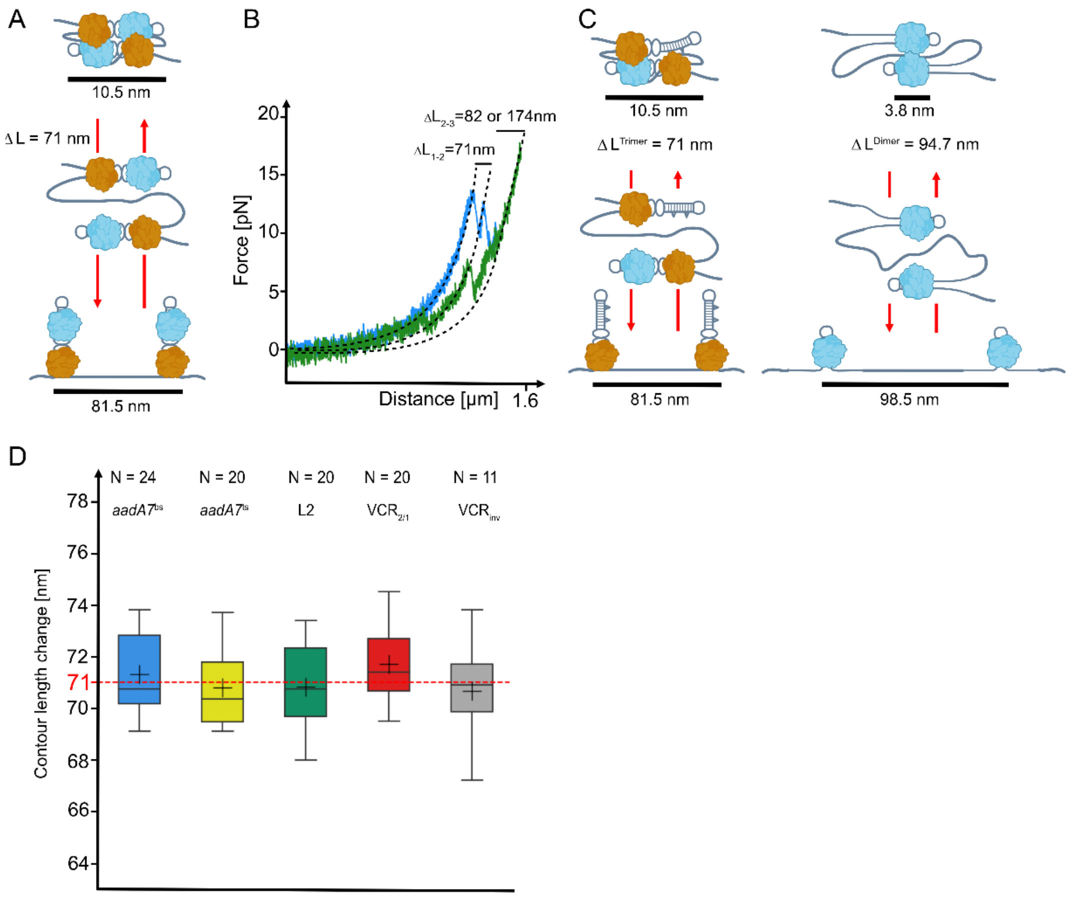

Fig. S3.

Scheme of the synaptic complex disassembly molecular fingerprint on the double-*attC* tether. **(A)** Synaptic complex disassembly scheme with the characteristic contour length change of 71 nm. The L-box bound subunits (blue) can remain bound or detach after the synaptic complex disassembly (indicated with semi-transparent color). **(B)** Characteristic force-extension curves for high (in blue) and low (in green) recombination efficiency *attC* sites. Synaptic complex disassembly is identified using an established molecular fingerprint $\Delta L_{1\text{-}2} = 71$ nm (experimental data $\Delta L_{1\text{-}2} = 71.1 \pm 1.6$ nm, N = 95), and the characteristic force of the event is recorded using the breaking point of the state (1) on the force-extension curve. Subsequent events of hairpins unfolding produce the theoretical $\Delta L_{2\text{-}3} = 82$ nm for short *attC*s or 174 nm for VCR *attC* sites (experimental data $\Delta L_{2\text{-}3} = 83 \pm 2$ nm, N = 11 and 174 ± 1 nm, N = 5). **(C)** Theoretical trimer and dimer formation scheme of double-*attC* complexes with calculated contour length change upon disassembly. A trimer-bound synaptic-like assembly lacking the L-box bound subunit would produce the same $\Delta L_{1\text{-}2} = 71$ nm as a correctly formed synaptic complex and would be indistinguishable in our assay. A dimer-bound complex, if possible at all, would result in $\Delta L_{1\text{-}2} = 98.5$ nm which is much higher than the established molecular fingerprint of the synaptic complex disassembly and would not be identified as a synaptic complex by our assay. **(D)** Box-plot of the contour length change upon synaptic complex disassembly for different *attC* constructs. The mean for each dataset is indicated with a black cross, the amount of datapoints and the name of the dataset is given above. The calculated contour length change of 71 nm is indicated with red dashed line.

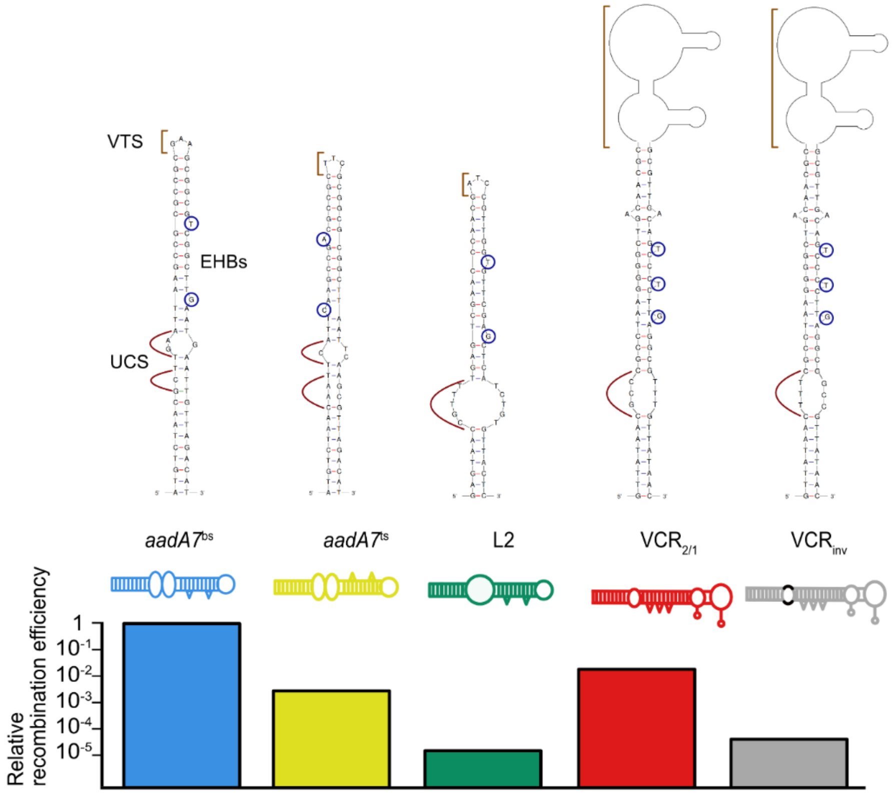

Fig. S4.

Sequence and folding architecture of the investigated *attC* sites depicted using Mfold (*48*). Structural parts are indicated: UCS with red lines, EHBs with blue circles, and VTS with brown lines. Relative recombination efficiency of the integration *in vivo (10, 11, 13, 20, 23)*.

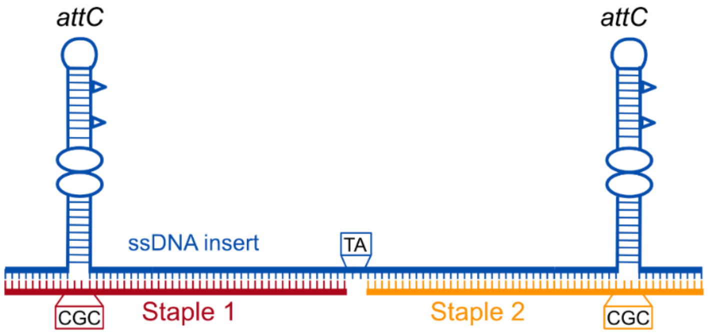

Fig. S5.

EMSA sample preparation design to minimize the amount of ssDNA. We stabilized the hairpin structure using two long complementary oligonucleotides (staple 1 and staple 2). CGC sequence was inserted to accommodate the width of the hairpin, TA bases in the middle of the spacer were left unpaired to allow the spacer necessary flexibility for synaptic complex assembly.

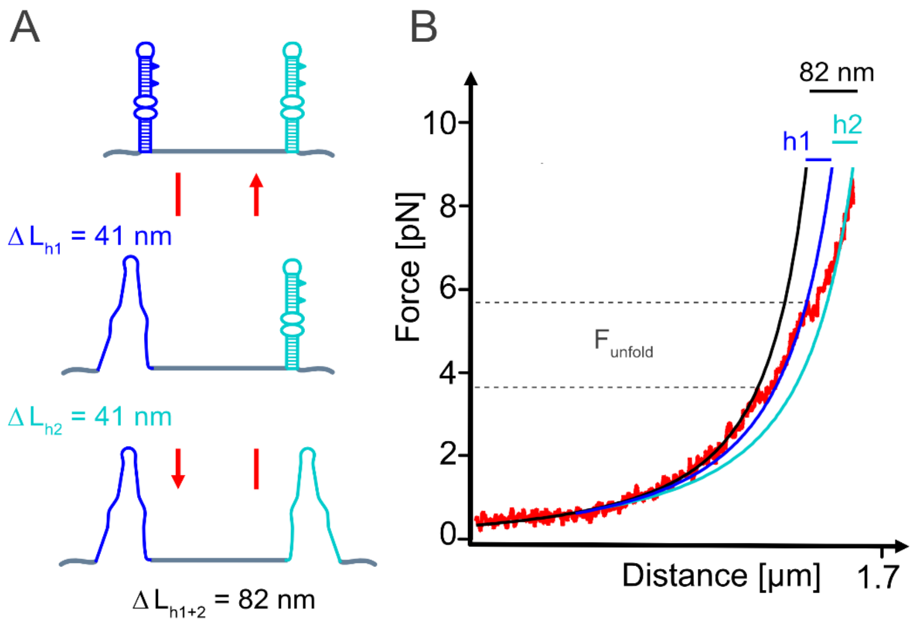

Fig. S6.

Scheme of the double-*attC* tether unfolding behavior in the absence of IntI. (A) Double-hairpin unfolding scheme achieved through two consecutive steps of single hairpin unfolding with the characteristic contour length change of 41 nm per hairpin (ΔL_{h1} and ΔL_{h2}). **(B)** Characteristic force-extension curves for *attC* hairpin unfolding and the characteristic force of the events is indicated with grey dashed lines.

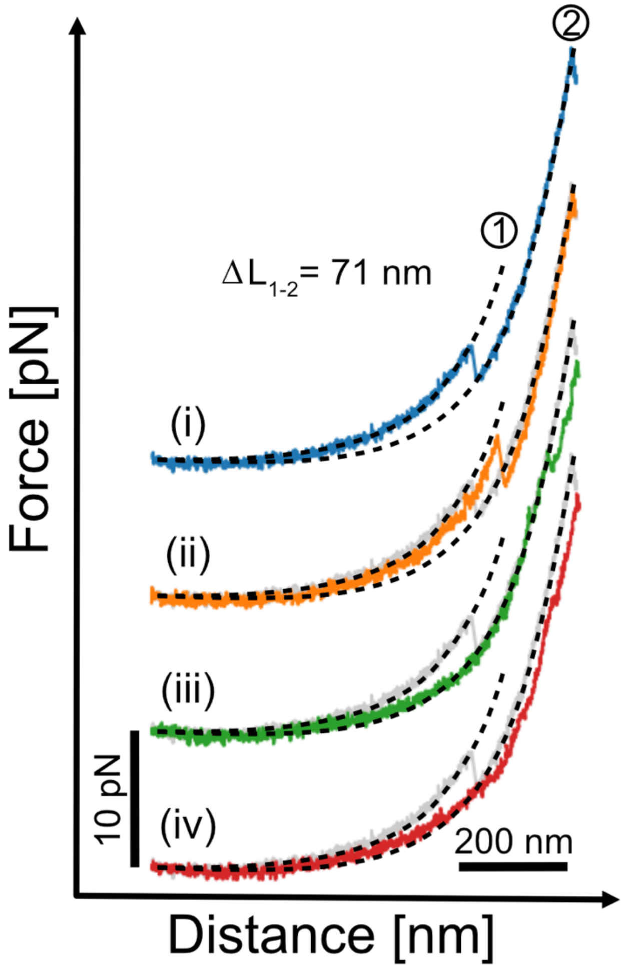

Fig. S7.

Force-extension curves of four consecutive stretching cycles of a double-*attC*$_{aadA7}$ ^{bs} tether. After incubation in the protein channel, the tether was moved to the buffer channel. The first stretching cycle results in synaptic complex disassembly, identified by the molecular finger print of 71 nm. The second stretching shows a mechanical stable structure, yet with a short contour length increase, likely a non-canonical state, but no well-defined synaptic complex. The third and fourth stretching cycles start at State 2 (folded *attC* hairpins separated by the spacer) and show only hairpin unfolding events upon stretching. Graphs are vertically offset for better visibility. The grey curve represents the disassembly event of cycle 1 for reference.

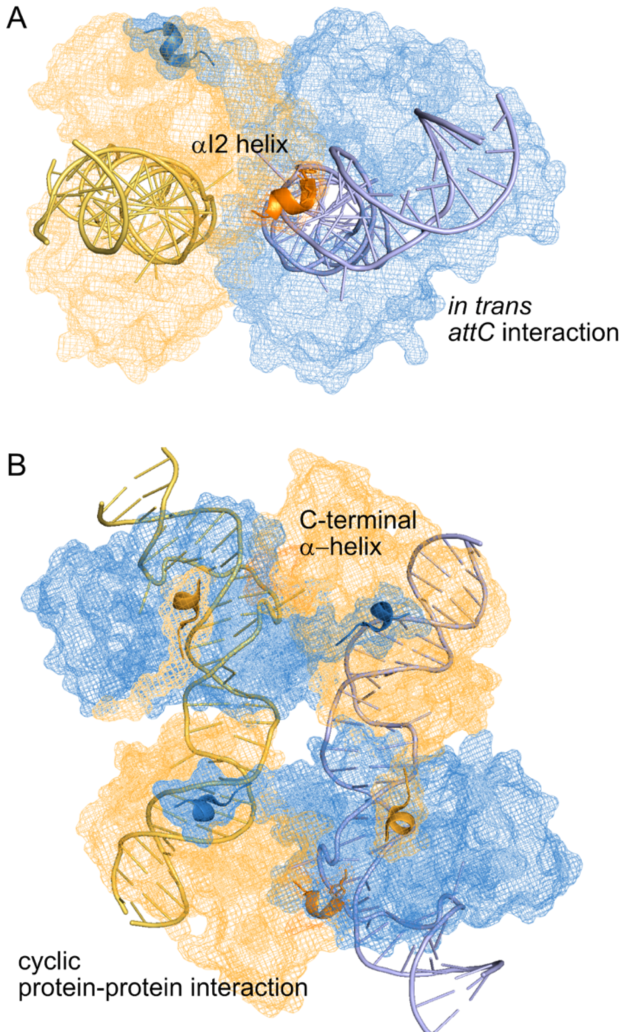

Fig. S8.

Protein-DNA and protein-protein interactions that are specific only for the synaptic complex formation. (A) αI2 helix interaction with adjacent DNA backbone *in trans* by K^{209} and Y^{210} (numbering according to IntI4 resolved synapse, (*19*)). **(B)** C-terminal α-helices that cyclically connect subunits in a tetramer. Visualized with PyMOL (*56*)

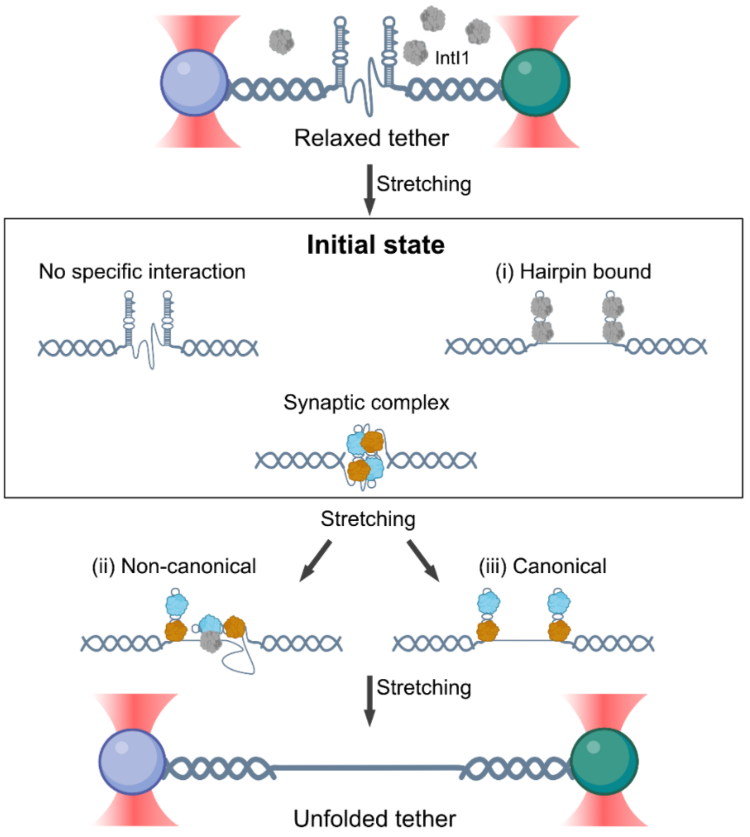

Fig. S9.

Schematic representation of possible integrase interaction modes with the double-*attC* tether. Three possible initial states resulting from integrase interaction with the tether are depicted in a frame: no specific interaction, (i) hairpin-bound or synaptic complex. Synaptic complex initial state can further disassemble via two pathways leading to (ii) non-canonical or (iii) canonical structures.

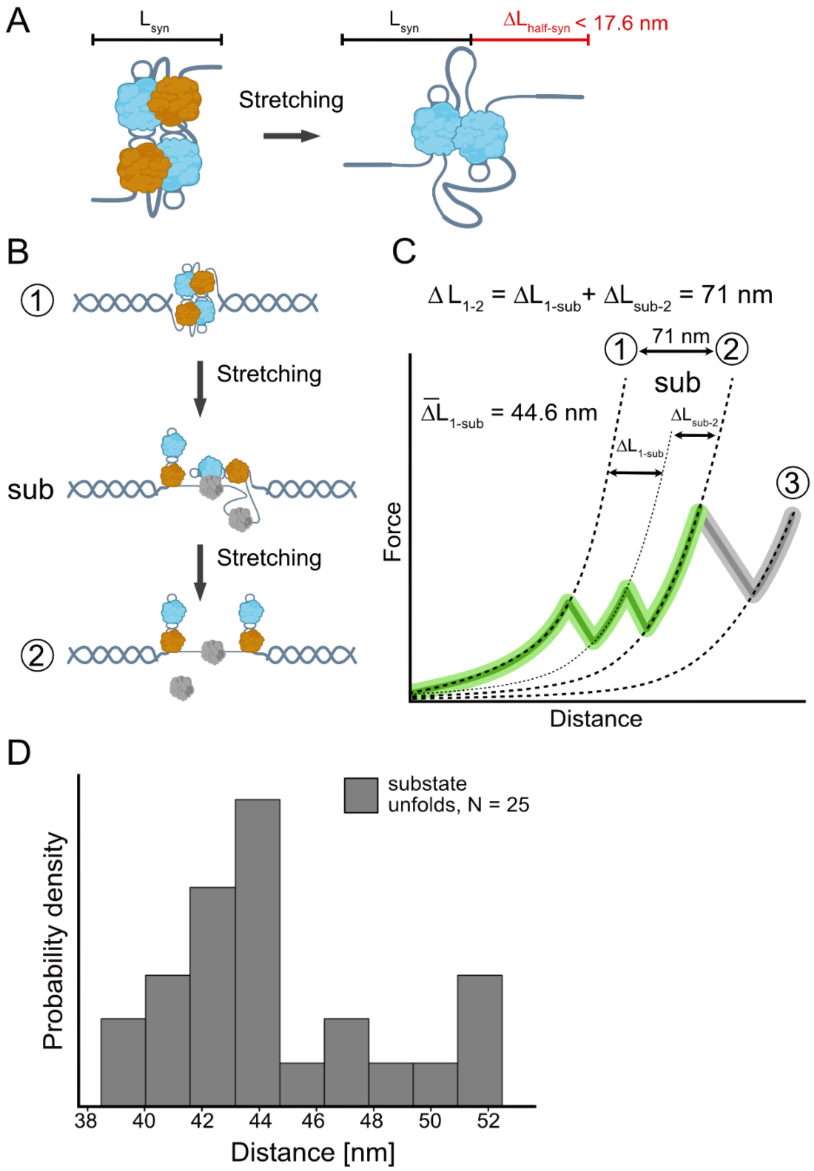

Fig. S10.

A scheme of possible non-canonical substate structural formations. (A) A schematic representation of the maximum contour length change possible for a semi-formed synaptic complex (half-syn). The $\Delta L < 17.6$ nm is the maximum value achieved by unfolding both R-boxes (26 nts). **(B)** A schematic representation of the proposed non-canonical pathway substate structure formation upon the stretching of the tether. **(C)** A force-extension curve depicting the substate and the mean contour length change to the substate ($\Delta L_{1\text{-}sub}$) and from the substate to the hairpin-bound state ($\Delta L_{sub\text{-}2}$). **(D)** A histogram of the contour length change for non-canonical unfolding events that led to the substate structure ($\Delta L_{1\text{-}sub}$), sampled from different *attC* sites.

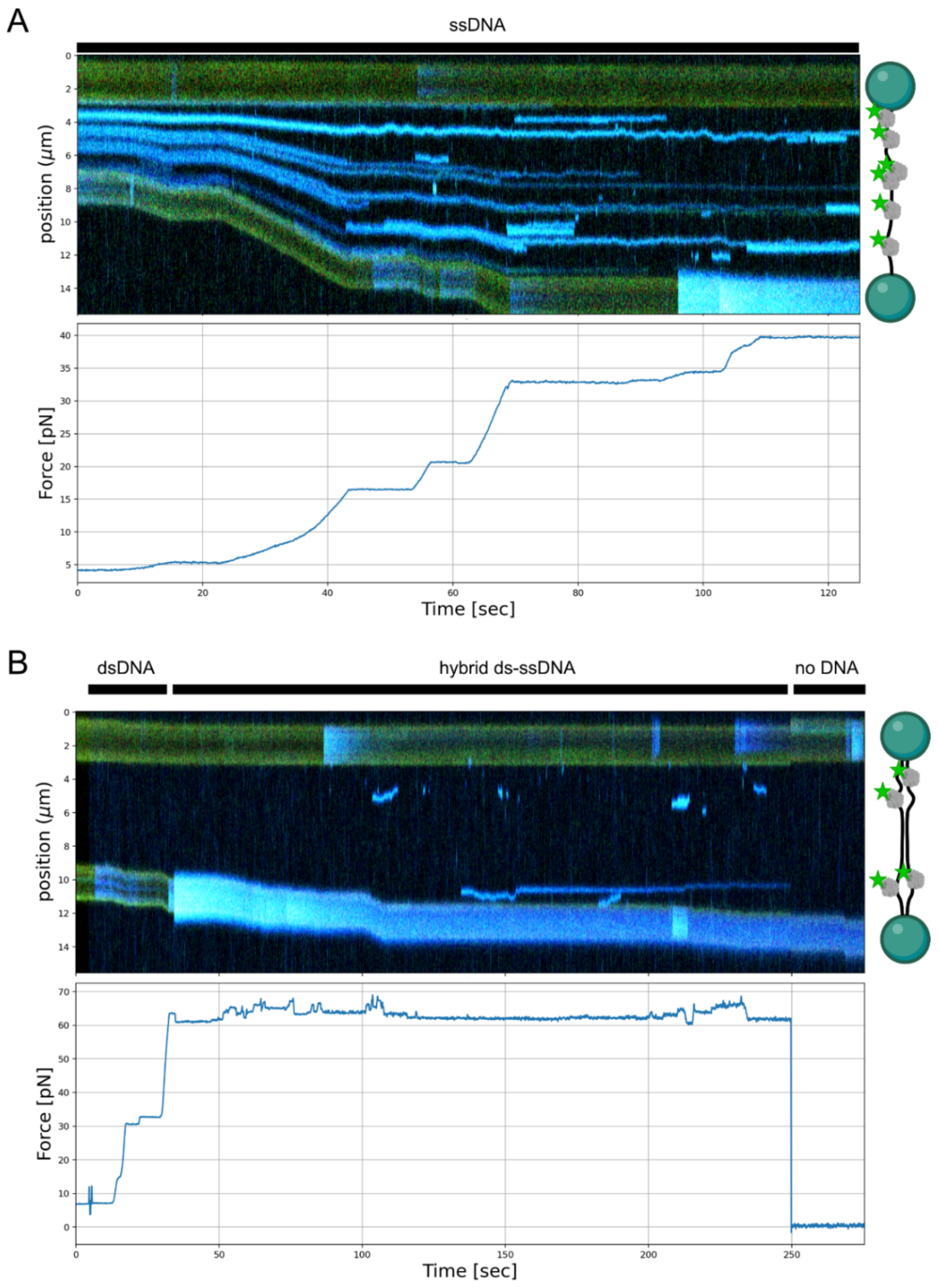

Fig. S11.

Kymographs and corresponding forces of the ds-ssDNA tethers in the presence of IntI1$_{\mathrm{mEGFP}}$. **(A)** ssDNA tether is stretched from 5 to 40 pN in the presence of IntI1$_{\mathrm{mEGFP}}$. **(B)** The dsDNA molecule is overstretched to a hybrid ds-ssDNA state in the presence of IntI1$_{\mathrm{mEGFP}}$. The blue signal indicates integrase binding.

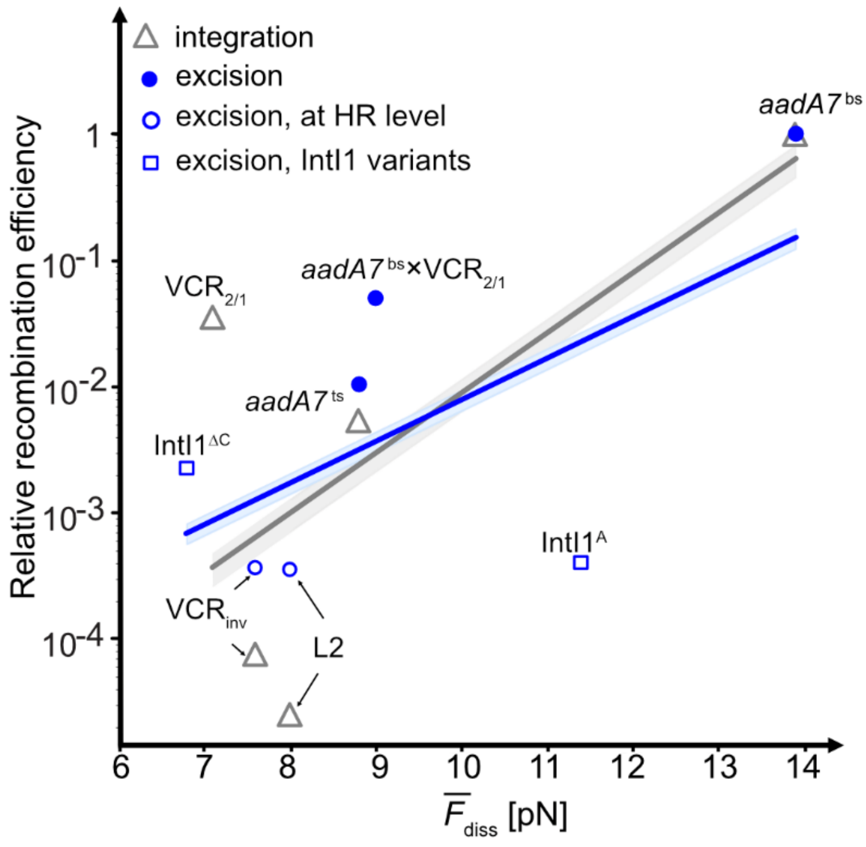

Fig. S12.

Correlation plot for recombination efficiency and mechanical stability of the synaptic complex. Relative recombination efficiency and mean synaptic complex disassembly force showing two sets of data: excision recombination efficiency measured in this study (blue) for different *attC* sites and integrase variants and integration recombination efficiency based on literature data *(10, 11, 13, 22, 23)* hypothetically correlated with forces measured for excision constructs (grey triangles). Different *attC* sites are indicated with circles, IntI1 variants with squares. Filled circles indicate that obtained recombination efficiency was IntI1-mediated, whence empty circles and squares show efficiency at the level of homology recombination. Integration recombination efficiencies are displaced from the correlation stronger, likely due to slightly different complex structure with *attI* × *attC* combination and potentially different mechanical stabilities. Yet, the overall trend of low recombination efficiency and lower mechanical stability is conserved. Protein mutations destabilize the synaptic complex to different extents and biological activity is even more diminished. Correlation fits include either all blue data points of excision synaptic complexes (blue line) or all triangle-indicated *attC* sites of integration reaction (grey fit).

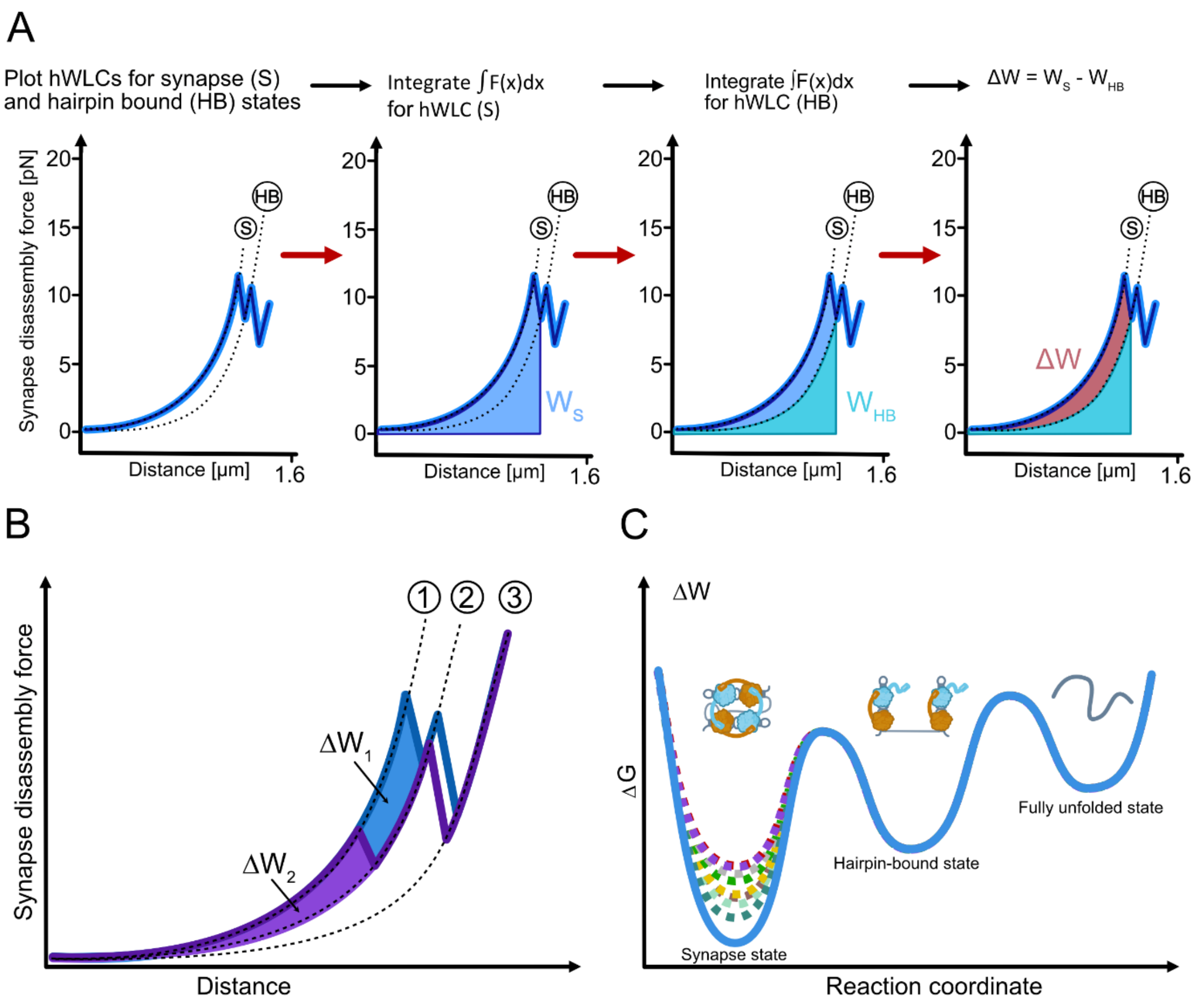

Fig. S13.

Mechanical work calculations and free energy profile as a model representation of varying synaptic complex stability between different *attC* sites and protein variants. (A) Schematic representation of synaptic complex mechanical work calculation (ΔW). First, we plotted the hWLC of the synapse and hairpin-bound states, then we integrated the area under the synapse state curve – W_S. Next, we integrated the area under the hairpin-bound state curve – W_{HB} and subtracted it from the W_S to get only the mechanical work done by the tether upon synaptic complex disassembly – ΔW. **(B)** Mechanical work of synaptic complex disassembly for high (ΔW_1) and low (ΔW_2) mechanical stability. **(C)** Free energy profile of transition between synaptic complex, hairpin-bound and fully unfolded state determined from the mean mechanical work upon synapse disassembly ($\overline{\Delta W}$) for $attC^{bs}_{aadA7}$ (blue), $attC^{ts}_{aadA7}$ (yellow), $attC_{L2}$ (green), $attC_{VCRinv}$ (grey), $attC^{bs}_{aadA7}$ × $attC_{VCR2/1}$ (brown), $attC^{bs}_{aadA7}$ × $attC_{L2}$ (mint), IntI1$^{\Delta C}$ (purple), IntI1^{A} (teal).

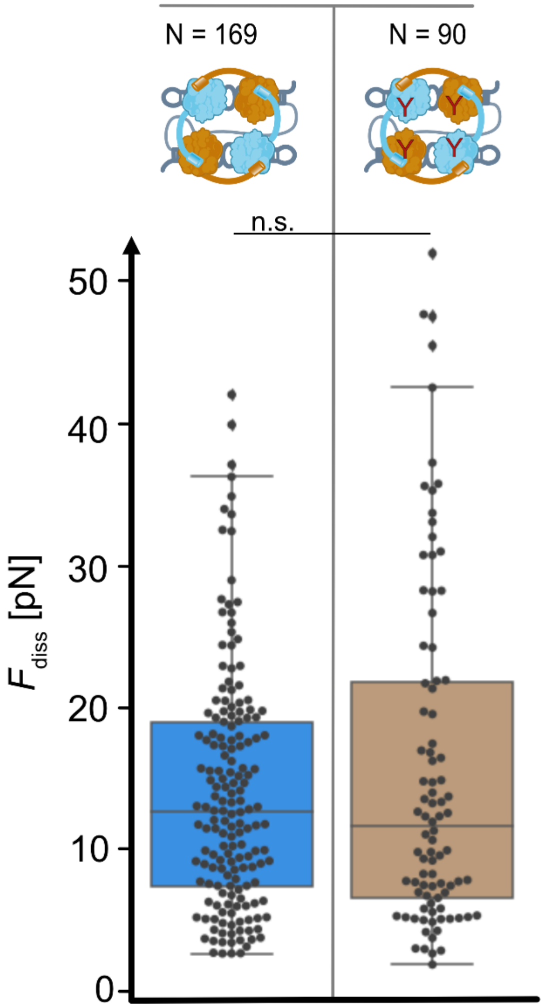

Fig. S14.

Mechanical stability of the synaptic complex formed with catalytically active and inactive IntI1. Boxplots of synaptic complex disassembly force for IntI:$attC_{aadA7}^{bs}$ synapse formed with IntI1^{Y312F} and IntI1^{wt} show no significant difference between the datasets. The pictograms for IntI1^{wt} is shown with the "Y" letter referring to the catalytic tyrosine residue.

Table S1.

Relative recombination efficiencies *in vivo* and synapse disassembly force for investigated IntI1:double-*attC* combinations. Relative recombination efficiency of the integration events for $attC_{aadA7}$ (both strands) is taken from (*11*, *13*), for $attC_{L2}$ from *(20)*, for $attC_{VCR2/1}$ from (*11*), for $attC_{VCR\mathrm{inv}}$ from *(10, 23)*; nr – no IntI1-mediated recombination. Calculated median, mean force, and the SEM (standard error of the mean) are presented for all synaptic complex disassembly events.

attC / IntI1 variant	*aadA7* ^{bs}	*aadA7* ^{ts}	$VCR_{2/1}$	*aadA7* ^{bs} × $VCR_{2/1}$	L2	VCR_{inv}	*aadA7* ^{bs} × L2
	IntI1						
Relative recombination efficiency (integration)	1	5.38×10^{-3}	3.55×10^{-2}	-	2.5×10^{-5}	7.56×10^{-5}	-
Relative recombination efficiency (excision)	1	1.03×10^{-2}	-	4.96×10^{-2}	3.55×10^{-4}	3.69×10^{-4}	nr
Median force (pN)	12.7	8.2	6.3	7.9	7.5	7.1	9.4
Mean force (pN)	13.9	8.8	7.1	9.0	8.0	7.6	11.4
SEM (pN)	0.6	0.4	0.3	0.4	0.3	0.4	0.6

attC / IntI1 variant	*aadA7* ^{bs}		
	$IntI1^{A}$	$IntI1^{\Delta C}$	$IntI1^{wt}$
Relative recombination efficiency (integration)	-	-	-
Relative recombination efficiency (excision)	nr	nr	-
Median force (pN)	9.4	5.6	11.6
Mean force (pN)	11.4	6.8	15.6
SEM (pN)	0.6	0.4	1.3

Table S2.

Table of all measurement data. The data collected in the integrase-filled channel is indicated as "IntI channel", non-specific integrase interaction events are indicated as well as the hairpin-bound events (HB) and synaptic complex disassembly events (ScD).

Protein	*attC*	# specific tethers	# traces with data (IntI channel)
IntI1	*aadA7*^{bs}	29	313
	aadA7^{ts}	23	400
	L2	22	314
	VCR$_{2/1}$	22	251
	aadA7^{bs} × VCR$_{2/1}$	2	324
	VCR$_{inv}$	19	155
	aadA7^{bs} × L2	4	306
IntI1^{A}	*aadA7*^{bs}	19	380
IntI1$^{\Delta C}$	*aadA7*^{bs}	25	385
IntI1^{wt}	*aadA7*^{bs}	5	188

Protein	*attC*	All channels	IntI channel				
		ScD	ScD			Non-specific	HB
			Total	Canonical	Non-canonical		
IntI1	*aadA7*^{bs}	169	128	95	33	180	55
	aadA7^{ts}	175	163	70	93	207	30
	L2	151	146	57	89	156	12
	VCR$_{2/1}$	126	124	50	74	104	22
	aadA7^{bs} × VCR$_{2/1}$	214	214	60	154	65	45
	VCR$_{inv}$	75	74	12	62	71	10
	aadA7^{bs} × L2	200	200	65	135	106	50
IntI1^{A}	*aadA7*^{bs}	144	141	25	116	169	70
IntI1$^{\Delta C}$	*aadA7*^{bs}	139	129	44	85	195	61
IntI1^{wt}	*aadA7*^{bs}	90	90	46	44	83	15

Table S3.

DNA sequences used in this study. (A) Primers for dsDNA handles. **(B)** Primers for dsDNA insert. **(C)** Primers for protein mutagenesis. **(D)** Primers and oligonucleotides for *in vivo* studies. **(E)** Full double-*attC* insert sequences. **(F)** Staple oligonucleotides for EMSA. **(G)** Plasmids. **(H)** Bacterial strains for *in vivo* conjugation studies. **(I)** Constructed plasmids for *in vivo* conjugation studies.

A. Primers designed for PCR amplification of dsDNA handles for double-*attC* constructs. Sequences are given in a 5' to 3' direction.

**Biotin functionalized dsDNA handle from lambda-DNA template**	
biotin_fwd_852bp_handle_AM	[Biotin] - CAGCATTGGTGACCTTGTTC
bio_rev_2500bp_primer	GGTATAGCATTCTAGCATTCAGCAGCACTTTAAACTGTCG
**Triple-digoxigenin functionalized dsDNA handle from lambda-DNA template**	
Triple-dig_fwd (70)	[Triple-DIG] - ATCCGCAGAAGACGCAGATGCC
3Dig_rev_2500bp_primer	ACGCAATGCGCAATGCAGGTCTTTTTCTGCTCTGACATGACG

B. Primers designed for PCR amplification of dsDNA insert sequence using a corresponding plasmid template. 5'- phosphorylation is shown as [Phos]. Sequences are given in a 5' to 3' direction.

**Insert with double-$attC_{aadA7}^{bs}$; double-$attC_{aadA7}^{ts}$**	
db_aadA7_few_sh	CATTGCGCATTGCGTAGATCTTTTATG
db_aadA7_rev_sh_phos	[Phos] - CTAGAATGCTATACCCTCGAGAAAATGTC
**Insert with double-$attC_{L2}$; double-$attC_{VCR2/1:}$ double-$attC_{VCRinv}$**	
Fwd_VCR	CATTGCGCATTGCGTAGATCTTTTG
Rev_VCR_phos	[Phos] - CTAGAATGCTATACCCTCGAGAAAG

C. Primers for protein mutagenesis. Sequences are given in a 5' to 3' direction

**Protein mutagenesis using QuikChange (Agilent)**	
primer_fwd_mut_stop	GTGCTGAAAGTTGGCGGTTAGTGAGTGCGCTCACCGCTTG
primer_fwd_mut_Ala	ACGCCCTTGAGCGGGCGGCTCCGCGCGCCGGGC

D. Primers and oligonucleotides used for *in vivo* studies. Sequences are given in a 5' to 3' direction

7166	CGTCGATGAAGATGAATTTTCTGGCG
7167	CGCCAGAAAATTCATCTTCATCGACG
7168	GCAATGGCATCCTGGTCATCCAGCGG
7169	CCGCTGGATGACCAGGATGCCATTGC
7223	TGAGGCAGCGCAAGTCAATCCTGGCGGATTCACTACCCCTGCGCGAAGGC
7229	AATTCGGTTATAACGGCCGCCTCAAGAGGGACTGTCAACGCGTGGCGTTTCCAGTCCCA TTGAGCCGCGGTGG
7230	TTGCTGTTGTTGTGTTTGAGTTTAGTGGTAGTGCGTTGTCAGCCCCTTAGGCGAAAGTT AGATGG
7231	GAAACGCCACGCGTTGACAGTCCCTCTTGAGGCGGCCGTTATAACCG
7232	GATCCCATCTAACTTTCGCCTAAGGGGCTGACAACGCACTACCACTAAACTCAAACACA ACAACAGCAACCACCGCGGCTCAATGGGACTG
7233	CGGATCGTTGGGTTCGACTCAAAACGGTTACTCGGTATTTGCATGCTTACAGGAGC
7234	TTGGTGTTCGAGCTCATCTGTGTTACTCGATGGTCTAGAGGCGAAGC
7235	AATTCGGTTAGAGTAACACAGATGAGCTCGAACACCAACGGATCGTTGG GTTCGACTCAAAACGGTTACTCGATGG
7236	GATCCCATCGAGTAACCGTTTTGAGTCGAACCCAACGATCCGTTGGTGTT CGAGCTCATCTGTGTTACTCTAACCG
7237	CGCGGCTCAATGGGACTGGAAACGCCACGCGTTGACAGTCCCTCTTGAGG CGGCCGTTAGATGGTCTAGAGGCGAAGCGGC
7238	GTGGTTGCTGTTGTTGTGTTTGAGTTTAGTGGTAGTGCGTTGTCAGCCCCT TAGGCGAAAGTTAAGGTATTTGCATGCTTACAGGAGC
7245	CTAACCGCCAACTTTCAGCACATGCGTGTAAATCATCGTCGTAGAGACGT CGG
7246	GCCCGCTCAAGGGCGTCGGGAAGCGC
7247	GGCTCCGCGCGCCGGGCATTCCTGGC
Swbeg	CCGTCACAGGTATTTATTCGGCG
Swend	CCTCACTAAAGGGAACAAAAGCTG

E. Designed sequences of the insert in pUC57-Kan-Simple carrying vector amplified with the primers above. The sequence of an *attC* site is indicated in red; three dT are in grey; restriction sites flanking the hairpins are in green; ligation parts to the nicked handles are shown in blue and underlined. Sequences are given in a 5’ to 3’ direction.

$attC_{aadA7}^{bs}$	CATTGCGCATTGCGTAGATCTTTTATGTCTAACGCTTGAATTAAGCCGCGCCGCGAAGCGGCGTCGGCTTG<br>AATGAATTGTTAGACATTTTCCATGGCTATATTTCAGCATCATCACATCATCATCATCATCATCACAGGGT<br>AGATCATCATCATCAACACGGGACGACAGCAAATGGCACCCTTCTATAAGCTTTTTATGTCTAACGCTTGA<br>ATTAAGCCGCGCCGCGAAGCGGCGTCGGCTTGAATGAATTGTTAGACATTTTCTCGAGGGTATAGCATTCT<br>AG
$attC_{aadA7}^{ts}$	CATTGCGCATTGCGTAGATCTTTTATGTCTAACAATTCATTCAAGCCGACGCCGCTTCGCGGCGCGGCTTA<br>ATTCAAGCGTTAGACATTTTCCATGGCTATATTTCAGCATCATCACATCATCATCATCATCATCACAGGGT<br>AGATCATCATCATCAACACGGGACGACAGCAAATGGCACCCTTCTATAAGCTTTTTATGTCTAACAATTCA<br>TTCAAGCCGACGCCGCTTCGCGGCGCGGCTTAATTCAAGCGTTAGACATTTTCTCGAGGGTATAGCATTCT<br>AG
$attC_{L2}$	CATTGCGCATTGCGTAGATCTTTTGAGTAACCGTTTTGAGTCGAACCCAACGATCCGTTGGTGTTCGAGCT<br>CATCTGTGTTACTCTTTCCATGGCTATATTTCAGCATCATCACATCATCATCATCATCATCACAGGGTAGA<br>TCATCATCATCAACACGGGACGACAGCAAATGGCACCCTTCTATAAGCTTTTTGAGTAACCGTTTTGAGTC<br>GAACCCAACGATCCGTTGGTGTTCGAGCTCATCTGTGTTACTCTTTCTCGAGGGTATAGCATTCTAG
$attC_{VCR2/1}$	CATTGCGCATTGCGTAGATCTTTTGTTATAACGCCCGCCTAAGGGGCTGACAACGCACTACCACTAAACTC<br>AAACACAACAACAGCAACCACCGCGGCTCAATGGGACTGGAAACGCCACGCGTTGACAGTCCCTCTTGAGG<br>CGTTTGTTATAACTTTCCATGGCTATATTTCAGCATCATCACATCATCATCATCATCATCACAGGGTAGAT<br>CATCATCATCAACACGGGACGACAGCAAATGGCACCCTTCTATAAGCTTTTTGTTATAACGCCCGCCTAAG<br>GGGCTGACAACGCACTACCACTAAACTCAAACACAACAACAGCAACCACCGCGGCTCAATGGGACTGGAAA<br>CGCCACGCGTTGACAGTCCCTCTTGAGGCGTTTGTTATAACTTTCTCGAGGGTATAGCATTCTAG
$attC_{VCRinv}$	CATTGCGCATTGCGTAGATCTTTTGTTATAACTTTCGCCTAAGGGGCTGACAACGCACTACCACTAAACTC<br>AAACACAACAACAGCAACCACCGCGGCTCAATGGGACTGGAAACGCCACGCGTTGACAGTCCCTCTTGAGG<br>CGGCCGTTATAACTTTCCATGGCTATATTTCAGCATCATCACATCATCATCATCATCATCACAGGGTAGAT<br>CATCATCATCAACACGGGACGACAGCAAATGGCACCCTTCTATAAGCTTTTTGTTATAACTTTCGCCTAAG<br>GGGCTGACAACGCACTACCACTAAACTCAAACACAACAACAGCAACCACCGCGGCTCAATGGGACTGGAAA<br>CGCCACGCGTTGACAGTCCCTCTTGAGGCGGCCGTTATAACTTTCTCGAGGGTATAGCATTCTAG
$attC_{aadA7}^{bs}$× $attC_{VCR2/1}$	CATTGCGCATTGCGTAGATCTTTTATGTCTAACGCTTGAATTAAGCCGCGCCGCGAAGCGGCGTCGGCTTG<br>AATGAATTGTTAGACATTTTCCATGGCTATATTTCAGCATCATCACATCATCATCATCATCATCACAGGGT<br>AGATCATCATCATCAACACGGGACGACAGCAAATGGCACCCTTCTATAAGCTTTTTGTTATAACGCCCGCC<br>TAAGGGGCTGACAACGCACTACCACTAAACTCAAACACAACAACAGCAACCACCGCGGCTCAATGGGACTG<br>GAAACGCCACGCGTTGACAGTCCCTCTTGAGGCGTTTGTTATAACTTTCTCGAGGGTATAGCATTCTAG
$attC_{aadA7}^{bs}$× $attC_{L2}$	CATTGCGCATTGCGTAGATCTTTTATGTCTAACGCTTGAATTAAGCCGCGCCGCGAAGCGGCGTCGGCTTG<br>AATGAATTGTTAGACATTTTCCATGGCTATATTTCAGCATCATCACATCATCATCATCATCATCACAGGGT<br>AGATCATCATCATCAACACGGGACGACAGCAAATGGCACCCTTCTATAAGCTTTTTGAGTAACCGTTTTGA<br>GTCGAACCCAACGATCCGTTGGTGTTCGAGCTCATCTGTGTTACTCTTTCTCGAGGGTATAGCATTCTAG

F. Staple oligonucleotides for EMSA. Sequences are given in a 5' to 3' direction.

Staple 1	CCCTGTGATGATGATGATGATGATGTGATGATGCTGAAATATAGCCATGGACGCAGA TCTACGCAATGCGCAATG
Staple 2	CTAGAATGCTATACCCTCGAGCGCAAGCTTATAGAAGGGTGCCATTTGCTGTCGTCC CGTGTTGATGATGATGATC

G. Plasmids used in this study.

**Plasmid name**	**Description**	**Reference**
**Template plasmids for double-*attC* construct**		
double_aadA7_lessCAT_pUC57-Kan-Simple	Containing double-$attC_{aadA7}^{bs}$	This study
aadA7-ts_original seq_pUC57-Kan-Simple	Containing double-$attC_{aadA7}^{ts}$	This study
aadA7-L2_original seq_pUC57-Kan-Simple	Containing double-$attC_{L2}$	This study
VCR-wt_original seq_pUC57-Kan-Simple	Containing double-$attC_{VCR2/1}$	This study
VCR-inv_original seq_pUC57-Kan-Simple	Containing double-$attC_{VCRinv}$	This study
**Plasmids containing MBP-IntI1**		
pMAL_c5X_IntI_Y312F_MBP	Catalytically inactive MBP-IntI1^{Y312F} expression plasmid	(*25*)
pMAL_c5X_IntI_Y312F_K219A_Y220A_MBP	Catalytically inactive MBP-IntI1^{A} expression plasmid	This study
pMAL_c5X_IntI_Y312F_A321X_G322X_MBP	Catalytically inactive MBP-IntI1$^{\Delta C}$ expression plasmid	This study
pMAL_c5X_IntI_Y312F_mEGFP_MBP	Catalytically inactive MBP-mEGFP-IntI1^{Y312F} expression plasmid	This study
pMAL_c5X_IntI_wt_MBP	Catalytically active MBP-IntI1^{wt} expression plasmid	(*25*)

H. Bacterial strains for *in vivo* conjugation studies.

**Plasmid name**	**Description**	**Reference**
**Receptor strain for conjugation**		
V141-V143	MG1655 Δ*recA* (4826) pL290	This study
V144-V146	MG1655 Δ*recA* (4826) pL294	This study
V931-V936	MG1655 Δ*recA* (4826) pV807- pV809	This study
V937-V942	MG1655 Δ*recA* (4826) pV810- pV813	This study
**Donor strain for conjugation**		
V147-V149	β2163Δ*km* pV068, pV069	This study
V971-V976	β2163Δ*km* pV552-pV554	This study

V977-V978	β2163Δ*km* pV557	This study
W869-W871	β2163Δ*km* pW654-pW656	This study
W872-W874	β2163Δ*km* pW657	This study
W863-W865	β2163Δ*km* p6944	This study
W866-W868	β2163Δ*km* pV967-pV969	This study

I. Constructed plasmids for *in vivo* conjugation studies.

**Plasmid name**	**Description**	**Notes**	**Reference**
pV068-pV069	pSW23T*::attC$_{aadA7}$-lacIq-attC$_{aadA7}$* (bs)	*ori$V_{R6K\gamma}$, oriT_{RP4}; [Cm^R]. The p6944 vector was amplified by PCR with 7169 and 7166 primers. The insert fragment was amplified by PCR from pG291 with 7167 and 7168. Assembly of these two fragments was achieved by performing Gibson Assembly*	This study
pV552-pV554	pSW23T*::attC$_{aadA7}$-lacIq-attC$_{aadA7}$* (bs)	*ori$V_{R6K\gamma}$, oriT_{RP4}; [Cm^R]. EcoRI/SalI digested fragment from pV068 cloned in EcoRI/SalI digested p4747*	This study
pV557	pSW23T*::attC$_{aadA7}$-lacIq-attC$_{aadA7}$* (ts)	*ori$V_{R6K\gamma}$, oriT_{RP4}; [Cm^R]. EcoRI/SalI digested fragment from pV068 cloned in EcoRI/SalI digested p4746*	This study
pV804-pV806	pSW23T*::attC$_{aadA7}$-lacIq-VCR$_{inv}$* (bs)	Amplification of p6944 by inverse PCR with primers 7237 and 7238	This study
pV967-pV969	pSW23T*::attC$_{aadA7}$-lacIq-L2* (bs)	Amplification of p6944 by inverse PCR with o7233 and o7234	This study
pW654-pW656	pSW23T*::VCR$_{inv}$-lacIq-VCR$_{inv}$* (bs)	Digestion of the pV806 plasmid by EcoRI/BamHI and insertion of the annealed primers o7229/o7230/o7231/o7232	This study
pW657	pSW23T*::L2-lacIq-L2* (bs)	Digestion of the pV969 plasmid by EcoRI/BamHI and insertion of the annealed primers o7235/o7236	This study
pV807-pV809	pSC101*::*int1 *mut.*	Amplification of pL294 by inverse PCR with o7246 and o7247	This study
pV810-pV813	pSC101*::*int1 *trunc.*	Amplification of pL294 by inverse PCR with o7223 and o7245	This study

Table S4.

Protein sequences used in this study. MBP tag is indicated in blue, IntI1^{Y312F} is indicated in yellow, mutated positions are indicated in red.

>MBP-IntI1^{Y312F} (IntI1)

MKIEEGKLVIWINGDKGYNGLAEVGKKFEKDTGIKVTVEHPDKLEEKFPQVAATGDGPDIIFWAHDRFGGYAQSGLLAEITPDK
AFQDKLYPFTWDAVRYNGKLIAYPIAVEALSLIYNKDLLPNPPKTWEEIPALDKELKAKGKSALMFNLQEPYFTWPLIAADGGY
AFKYENGKYDIKDVGVDNAGAKAGLTFLVDLIKNKHMNADTDYSIAEAAFNKGETAMTINGPWAWSNIDTSKVNYGVTVLPTFK
GQPSKPFVGVLSAGINAASPNKELAKEFLENYLLTDEGLEAVNKDKPLGAVALKSYEEELVKDPRIAATMENAQKGEIMPNIPQ
MSAFWYAVRTAVINAASGRQTVDEALKDAQTNSSSNNNNNNNNNNLGIEGRISHMSMGGRDIGRSENLYFQGSMKTATAPLPPL
RSVKVLDQLRERIRYLHYSLRTEQAYVHWVRAFIRFHGVRHPATLGSSEVEAFLSWLANERKVSVSTHRQALAALLFFYGKVLC
TDLPWLQEIGRPRPSRRLPVVLTPDEVVRILGFLEGEHRLFAQLLYGTGMRISEGLQLRVKDLDFDHGTIIVREGKGSKDRALM
LPESLAPSLREQLSRARAWWLKDQAEGRSGVALPDALERKYPRAGHSWPWFWVFAQHTHSTDPRSGVVRRHHMYDQTFQRAFKR
AVEQAGITKPATPHTLRHSFATALLRSGYDIRTVQDLLGHSDVSTTMIFTHVLKVGGAGVRSPLDALPPLTSER*

>MBP-IntI1^{Y312F_K219A_Y220A} (IntI1^{A})

MKIEEGKLVIWINGDKGYNGLAEVGKKFEKDTGIKVTVEHPDKLEEKFPQVAATGDGPDIIFWAHDRFGGYAQSGLLAEITPDK
AFQDKLYPFTWDAVRYNGKLIAYPIAVEALSLIYNKDLLPNPPKTWEEIPALDKELKAKGKSALMFNLQEPYFTWPLIAADGGY
AFKYENGKYDIKDVGVDNAGAKAGLTFLVDLIKNKHMNADTDYSIAEAAFNKGETAMTINGPWAWSNIDTSKVNYGVTVLPTFK
GQPSKPFVGVLSAGINAASPNKELAKEFLENYLLTDEGLEAVNKDKPLGAVALKSYEEELVKDPRIAATMENAQKGEIMPNIPQ
MSAFWYAVRTAVINAASGRQTVDEALKDAQTNSSSNNNNNNNNNNLGIEGRISHMSMGGRDIGRSENLYFQGSMKTATAPLPPL
RSVKVLDQLRERIRYLHYSLRTEQAYVHWVRAFIRFHGVRHPATLGSSEVEAFLSWLANERKVSVSTHRQALAALLFFYGKVLC
TDLPWLQEIGRPRPSRRLPVVLTPDEVVRILGFLEGEHRLFAQLLYGTGMRISEGLQLRVKDLDFDHGTIIVREGKGSKDRALM
LPESLAPSLREQLSRARAWWLKDQAEGRSGVALPDALERAAPRAGHSWPWFWVFAQHTHSTDPRSGVVRRHHMYDQTFQRAFKR
AVEQAGITKPATPHTLRHSFATALLRSGYDIRTVQDLLGHSDVSTTMIFTHVLKVGGAGVRSPLDALPPLTSER*

>MBP-IntI1^{Y312F_A321X_G322X} (IntI1$^{\Delta C}$)

MKIEEGKLVIWINGDKGYNGLAEVGKKFEKDTGIKVTVEHPDKLEEKFPQVAATGDGPDIIFWAHDRFGGYAQSGLLAEITPDK
AFQDKLYPFTWDAVRYNGKLIAYPIAVEALSLIYNKDLLPNPPKTWEEIPALDKELKAKGKSALMFNLQEPYFTWPLIAADGGY
AFKYENGKYDIKDVGVDNAGAKAGLTFLVDLIKNKHMNADTDYSIAEAAFNKGETAMTINGPWAWSNIDTSKVNYGVTVLPTFK
GQPSKPFVGVLSAGINAASPNKELAKEFLENYLLTDEGLEAVNKDKPLGAVALKSYEEELVKDPRIAATMENAQKGEIMPNIPQ
MSAFWYAVRTAVINAASGRQTVDEALKDAQTNSSSNNNNNNNNNNLGIEGRISHMSMGGRDIGRSENLYFQGSMKTATAPLPPL
RSVKVLDQLRERIRYLHYSLRTEQAYVHWVRAFIRFHGVRHPATLGSSEVEAFLSWLANERKVSVSTHRQALAALLFFYGKVLC
TDLPWLQEIGRPRPSRRLPVVLTPDEVVRILGFLEGEHRLFAQLLYGTGMRISEGLQLRVKDLDFDHGTIIVREGKGSKDRALM
LPESLAPSLREQLSRARAWWLKDQAEGRSGVALPDALERKYPRAGHSWPWFWVFAQHTHSTDPRSGVVRRHHMYDQTFQRAFKR
AVEQAGITKPATPHTLRHSFATALLRSGYDIRTVQDLLGHSDVSTTMIFTHVLKVGG**

>MBP-mEGFP-IntI1^{Y312F} (IntI1$_{mEGFP}$)

MKIEEGKLVIWINGDKGYNGLAEVGKKFEKDTGIKVTVEHPDKLEEKFPQVAATGDGPDIIFWAHDRFGGYAQSGLLAEITPDK
AFQDKLYPFTWDAVRYNGKLIAYPIAVEALSLIYNKDLLPNPPKTWEEIPALDKELKAKGKSALMFNLQEPYFTWPLIAADGGY

AFKYENGKYDIKDVGVDNAGAKAGLTFLVDLIKNKHMNADTDYSIAEAAFNKGETAMTINGPWAWSNIDTSKVNYGVTVLPTFK
GQPSKPFVGVLSAGINAASPNKELAKEFLENYLLTDEGLEAVNKDKPLGAVALKSYEEELVKDPRIAATMENAQKGEIMPNIPQ
MSAFWYAVRTAVINAASGRQTVDEALKDAQTNSSSMVSKGEELFTGVVPILVELDGDVNGHKFSVSGEGEGDATYGKLTLKFIC
TTGKLPVPWPTLVTTLTYGVQCFSRYPDHMKQHDFFKSAMPEGYVQERTIFFKDDGNYKTRAEVKFEGDTLVNRIELKGIDFKE
DGNILGHKLEYNYNSHNVYIMADKQKNGIKVNFKIRHNIEDGSVQLADHYQQNTPIGDGPVLLPDNHYLSTQSKLSKDPNEKRD
HMVLLEFVTAAGITLGMDELYKNNNNNNNNNNLGIEGRISHMSMGGRDIGRSENLYFQGSMKTATAPLPPLRSVKVLDQLRERI
RYLHYSLRTEQAYVHWVRAFIRFHGVRHPATLGSSEVEAFLSWLANERKVSVSTHRQALAALLFFYGKVLCTDLPWLQEIGRPR
PSRRLPVVLTPDEVVRILGFLEGEHRLFAQLLYGTGMRISEGLQLRVKDLDFDHGTIIVREGKGSKDRALMLPESLAPSLREQL
SRARAWWLKDQAEGRSGVALPDALERKYPRAGHSWPWFWVFAQHTHSTDPRSGVVRRHHMYDQTFQRAFKRAVEQAGITKPATP
HTLRHSFATALLRSGYDIRTVQDLLGHSDVSTTMIFTHVLKVGGAGVRSPLDALPPLTSER*

>MBP-IntI1^{wt} (IntI1^{wt})

MKIEEGKLVIWINGDKGYNGLAEVGKKFEKDTGIKVTVEHPDKLEEKFPQVAATGDGPDIIFWAHDRFGGYAQSGLLAEITPDK
AFQDKLYPFTWDAVRYNGKLIAYPIAVEALSLIYNKDLLPNPPKTWEEIPALDKELKAKGKSALMFNLQEPYFTWPLIAADGGY
AFKYENGKYDIKDVGVDNAGAKAGLTFLVDLIKNKHMNADTDYSIAEAAFNKGETAMTINGPWAWSNIDTSKVNYGVTVLPTFK
GQPSKPFVGVLSAGINAASPNKELAKEFLENYLLTDEGLEAVNKDKPLGAVALKSYEEELVKDPRIAATMENAQKGEIMPNIPQ
MSAFWYAVRTAVINAASGRQTVDEALKDAQTNSSSNNNNNNNNNNLGIEGRISHMSMGGRDIGRSENLYFQGSMKTATAPLPPL
RSVKVLDQLRERIRYLHYSLRTEQAYVHWVRAFIRFHGVRHPATLGSSEVEAFLSWLANERKVSVSTHRQALAALLFFYGKVLC
TDLPWLQEIGRPRPSRRLPVVLTPDEVVRILGFLEGEHRLFAQLLYGTGMRISEGLQLRVKDLDFDHGTIIVREGKGSKDRALM
LPESLAPSLREQLSRARAWWLKDQAEGRSGVALPDALERKYPRAGHSWPWFWVFAQHTHSTDPRSGVVRRHHMYDQTFQRAFKR
AVEQAGITKPATPHTLRHSFATALLRSGYDIRTVQDLLGHSDVSTTMIYTHVLKVGGAGVRSPLDALPPLTSER*

Table S5.
Python 3.10.5 packages used in this study.

lumicks.pylake 0.12.1	pathlib
numpy 1.23.1	Pandas 1.4.3
glob	Scipy 1.8.1
math	Seaborn 0.11.2
statistics	Pingouin 0.5.3
os	Statsmodels 0.13.2
Matplotlib 3.5.2	Sympy 1.12

Supplementary Protocols

Double-attC construct preparation

dsDNA handle preparation

PCR amplification of each dsDNA handle was performed in 50 μL volume using 20 ng of template lambda DNA (Promega, USA) using standard Taq polymerase protocol (New England Biolabs, USA) and the designed primer pair (Table S3A) at the annealing temperature of 54°C. The resulting PCR product was cleaned using standard ethanol precipitation protocol.

Nicking reaction

Each handle was nicked with a specific restriction enzyme separately according to the manufacturer's instructions (enzymes and buffers from New England Biolabs). The nicking reaction was performed in a thermocycler at 65°C for 1 hour, followed by the inactivation step of 80°C for 20 minutes. The resulting reaction product was cleaned using standard ethanol precipitation protocol.

dsDNA insert preparation

The template plasmids (Table S3G) containing the target double-*attC* sequences were commercially acquired (GenScript, USA) and transformed into XL-10 ultracompetent *E. coli* cells using standard heat-shock transformation protocol. The cells were plated on Kanamycin selection plates and grown according to the standard protocol. The resulting culture was used for plasmid DNA extraction using the NucleoSpin plasmid kit (Machery-Nagel, Germany) according to the manufacturer's instructions and eluted with nuclease-free water. PCR amplification of the insert sequence (Table S3E) was performed in 50 μL volume using 100 ng of template plasmid DNA using standard Taq polymerase protocol (New England Biolabs) and the designed primer pair (Table S3B). The resulting PCR product was cleaned using standard ethanol precipitation protocol.

Selective digestion for single-strand generation

To remove one strand from the dsDNA insert sequence we used 1 μL (5 units) of lambda exonuclease enzyme (New England Biolabs) on 2.5 μg dsDNA insert. The reaction mix was incubated at 37°C for 30 minutes, followed by the inactivation step of 80°C for 5 minutes.

Coupling reaction and ligation

First, one nicked dsDNA handle (5 pmol) is mixed with the ssDNA insert (5 pmol) and left for 15 minutes at room temperature for initial annealing. Then the second nicked dsDNA handle (5 pmol) is added to the mix and the tube is filled until 38 μL with nuclease-free water. The solution is rapidly heated up to 90°C and then slowly cooled down to 16°C for DNA strands annealing. After the coupling step, the reaction solution was supplied with T4 Ligase enzyme and buffer (Jena Bioscience) as well as extra ATP (to the final concentration of 4 mM) and kept at 16°C for 2 hours for ligation. The resulting hybrid DNA molecule was stored at 4°C and used for directly for optical tweezers experiments. All dsDNA products of the assembly were checked on 0.8% agarose gel (1× TBE buffer, running at 90V for 50 minutes) for quality control.

MBP-IntI1 variants mutagenesis and purification

Mutagenesis

The alanine mutant $IntI1^{A}$ was designed to substitute Lys^{219} and Tyr^{220} with alanines using a QuikChange kit (Agilent). Four nucleotides were substituted in the integrase gene sequence using "primer_fwd_mut_Ala" (Table S3C). The C-terminal truncated mutant $IntI1^{\Delta C}$ was designed to

substitute Ala^{321} and Gly^{322} to stop codons using the QuikChange kit (Agilent). Four nucleotides were substituted in the integrase gene sequence using "primer_fwd_mut_stop" (Table S3C).

Protein expression

We followed the protein expression and a two-step purification protocol previously developed in the lab (*25*). Each plasmid containing protein variant was transformed into the *E. coli* BL21 (DE3) strain using the heat-shock method and plated on agar containing 50 µg/mL ampicillin for overnight growth at 37°C. One colony was taken from the agar plate to start a 10 mL overnight culture with 50 µg/ml ampicillin at 37°C and 140 rpm. To start a culture 1 L of Terrific broth (TB) medium with 50 µg/mL ampicillin was inoculated using 5 mL of the overnight culture to grow at 37°C. When the culture reached an optical density = 0.6, protein expression was induced by adding isopropyl-D-1-thiogalactopyranoside (IPTG) to a final concentration of 0.3 mM. The induced culture was grown at 14°C overnight. The culture was centrifuged (Beckmann Coulter) at 5500 rpm for 30 min at 4°C, the pellet was collected.

Protein purification

Both purification steps were performed on the fast protein liquid chromatography (FPLC) device (ÄKTA Protein Purification System, Cytiva Life Sciences). The first step used the amylose column to separate MBP-tagged protein. However, this purification leaves also free MBP protein that presents as a defined band at 45 kDa on the SDS-PAGE gel. To remove the impurity a second purification step was introduced by using an ion exchange technique. Using difference in theoretical isoelectric points (pI) for the MBP and the MBP-IntI1 variants, separation by charge is possible with the buffer of appropriate pH.

1st step (MBPTrap).

The cell pellet was re-suspended in 60 mL Buffer A. Recovered cells were then lysed using EmulsiFlex-C3 (Avestin), and the resulting homogenized cells were centrifuged at 8400 rpm for 1 hour at 4 °C. Recovered lysate (supernatant) was filtered using 0.45 µm disposable filters to prevent column clogging. Filtered lysate was loaded onto the ÄKTA FPLC using the superloop at the flow rate of 1 mL/min. The unbound lysate was washed with 5 column volumes of BufferA with a flow rate of 1 mL/min. To elute the bound MBP-tagged protein the gradient from 0-100% of Buffer B relative to Buffer A was applied and the fractions of 1 mL were collected in a fraction collector.

Collected fractions were analyzed using the sodium dodecyl sulfate-polyacrylamide gel electrophoresis (SDS-PAGE) with 12% acrylamide hand-casted gel (Bio-Rad Minigel system). The gel was run at 150 V for 1 hour and then stained with Comassie by heating it for 30 seconds in the microwave. Afterward, the staining solution was removed and the de-staining solution was added (10% acidic acid) and left overnight on an orbital shaker. After de-staining was complete, the gel was scanned and the bands were analyzed.

2nd step (HiTrap).

Next, to remove the MBP impurity an ion exchange column was used on the same ÄKTA FPLC. The purified fractions were first dialyzed to exchange buffer. Using dialysis cassettes (20000 Da MWCO, Thermo Scientific) and placed in sufficient volume of Buffer C on the stirrer at 4°C. The buffer was changed after 2 hours and 3 hours and then left overnight. The dialyzed protein was diluted with Buffer D (1:4) before loading to lower salt concentration in the sample.

Dialysed fraction sample was loaded onto the FPLC using the superloop at the flow rate of 1 mL/min. The unbound lysate was washed with 5 column volumes of Buffer D with a flow rate of

1 mL/min. To elute the target protein, the gradient from 0-100% of Buffer E relative to Buffer D was applied, and the fractions of 1 mL were collected in a fraction collector. Collected fractions were analyzed using SDS-PAGE. High purity fractions were dialyzed to exchange the buffer using the same procedure described above to the final storage Buffer F. Protein concentration was calculated using Nanodrop measurements and the known extinction coefficient of the mutant proteins. The samples were aliquoted, frozen with liquid nitrogen, and placed at -70°C.

The buffers used were as follows:

- Buffer A (running buffer, amylose column): 50mM TRIS-HCl pH7.4, 1M NaCl, 10% glycerol
- Buffer B (elution buffer, amylose column): 50mM TRIS-HCl pH 7.4, 1M NaCl, 10mM maltose
- Buffer C (4× phosphate buffer exchange): 200mM phosphate buffer pH 7.2, 500mM NaCl, 30% glycerol
- Buffer D (running buffer, ion exchange column): 50mM phosphate buffer pH 7.2
- Buffer E (elution buffer, ion exchange column): 50mM phosphate buffer pH 7.2, 1M NaCl
- Buffer F (protein storage buffer): 50mM TRIS-HCl pH 7.4, 200mM NaCl, 30% glycerol

EMSA

We developed a protocol for non-radioactive EMSA using long ssDNA fragments and bulky fusion proteins that form large molecular complexes and present challenges using standard EMSA conditions. The gel is hand cast using the Bio-Rad Minigel system to produce 5% native polyacrylamide gel (acrylamide/bisacrylamide 37.5:1) with 0.5× TBE and 2.5% glycerol. The running buffer was mixed containing 0.5× TBE and 2.5% glycerol and used to pre-run the gel in the cold (4°C) at 80V for 60 minutes.

For optimal integrase-DNA binding conditions, we used the binding buffer (10 mM Tris-HCl, 10 mM NaCl, 40 mM KCl, 1 mM $MgCl_2$, 4 mM EDTA, 1 mM DTT, 0.5 mg/mL BSA, 5% glycerol). The ssDNA of the double-*attC* insert was mixed with the same amount (in pmol) of designed oligonucleotides (Staple 1 and Staple 2) in the final volume of 50 µL and placed in the thermocycler for *attC* hairpin formation and staple annealing (the mix was rapidly heated up to 95°C and then slowly cooled down).

The samples were assembled in 15 µL volume in the following order:

x µL mQ (15 µL – rest)
3 µL binding buffer (5x)
The required amount of IntI protein (diluted from stock with protein storage buffer)
x µL of DNA substrate (2 pmol) – after hairpin formation and staple annealing

The mixture was incubated at room temperature for 15 minutes, then 3 µL of OrangeG dye (6x) was added to the samples to track the run and the mixture was loaded on the gel. The electrophoresis was done in the cold (4°C) at 90 V for 120 minutes. After the run, the gel was placed in a closed box for staining using SYBRGold (Thermo Fisher Scientific) solution (5 µL stock in 50 mL running buffer) and left on the orbital shaker for 30 minutes in the dark. After staining was complete, the gel was rinsed using the running buffer and placed on the plastic film in the imager (Azure 300, Azure Biosystems). The gel was imaged with epi-blue light (LED, 472 nm) and the gel image was analyzed using Fiji.

REFERENCES AND NOTES

1. J. Davies, Inactivation of antibiotics and the dissemination of resistance genes. *Science* **264**, 375–382 (1994).

2. G. Cambray, A.-M. Guerout, D. Mazel, Integrons. *Annu. Rev. Genet.* **44**, 141–166 (2010).

3. D. Mazel, B. Dychinco, V. A. Webb, J. Davies, A distinctive class of integron in the Vibrio cholerae genome. *Science* **280**, 605–608 (1998).

4. D. A. Rowe-Magnus, A.-M. Guerout, D. Mazel, Bacterial resistance evolution by recruitment of super-integron gene cassettes. *Mol. Microbiol.* **43**, 1657–1669 (2002).

5. B. Sallen, A. Rajoharison, S. Desvarenne, C. Mabilat, Molecular epidemiology of integron-associated antibiotic resistance genes in clinical isolates of enterobacteriaceae. *Microb. Drug Resist.* **1**, 195–202 (1995).

6. S. Nandi, J. J. Maurer, C. Hofacre, A. O. Summers, Gram-positive bacteria are a major reservoir of Class 1 antibiotic resistance integrons in poultry litter. *Proc. Natl. Acad. Sci. U.S.A.* **101**, 7118–7122 (2004).

7. H. W. Stokes, R. M. Hall, A novel family of potentially mobile DNA elements encoding site-specific gene-integration functions: Integrons. *Mol. Microbiol.* **3**, 1669–1683 (1989).

8. Y. Lacotte, M.-C. Ploy, S. Raherison, Class 1 integrons are low-cost structures in *Escherichia coli*. *ISME J.* **11**, 1535–1544 (2017).

9. É. Guerin, G. Cambray, N. Sanchez-Alberola, S. Campoy, I. Erill, S. Da Re, B. Gonzalez-Zorn, J. Barbé, M.-C. Ploy, D. Mazel, The SOS response controls integron recombination. *Science* **324**, 1034–1034 (2009).

10. M. Bouvier, M. Ducos-Galand, C. Loot, D. Bikard, D. Mazel, Structural features of single-stranded integron cassette *attC* Sites and their role in strand selection. *PLOS Genet.* **5**, e1000632 (2009).

11. C. Loot, D. Bikard, A. Rachlin, D. Mazel, Cellular pathways controlling integron cassette site folding. *EMBO J.* **29**, 2623–2634 (2010).

12. C. Loot, A. Nivina, J. Cury, J. A. Escudero, M. Ducos-Galand, D. Bikard, E. P. C. Rocha, D. Mazel, Differences in integron cassette excision dynamics shape a trade-off between evolvability and genetic capacitance. *mBio* **8**, e02296-16 (2017).

13. A. Mukhortava, M. Pöge, M. S. Grieb, A. Nivina, C. Loot, D. Mazel, M. Schlierf, Structural heterogeneity of *attC* integron recombination sites revealed by optical tweezers. *Nucleic Acids Res.* **47**, 1861–1870 (2019).

14. H. Spåhr, T. Chia, J. P. Lingford, S. J. Siira, S. B. Cohen, A. Filipovska, O. Rackham, Modular ssDNA binding and inhibition of telomerase activity by designer PPR proteins. *Nat. Commun.* **9**, 2212 (2018).

15. B. Michel, After 30 years of study, the bacterial SOS response still surprises us. *PLoS Biol.* **3**, e255 (2005).

16. C. M. Collis, R. M. Hall, Gene cassettes from the insert region of integrons are excised as covalently closed circles. *Mol. Microbiol.* **6**, 2875–2885 (1992).

17. J. A. Escudero, C. Loot, A. Nivina, D. Mazel, The integron: Adaptation on demand. *Microbiol. Spectr.* **3**, MDNA3-0019-2014 (2015).

18. D. MacDonald, G. Demarre, M. Bouvier, D. Mazel, D. N. Gopaul, Structural basis for broad DNA-specificity in integron recombination. *Nature* **440**, 1157–1162 (2006).

19. M. Bouvier, G. Demarre, D. Mazel, Integron cassette insertion: A recombination process involving a folded single strand substrate. *EMBO J.* **24**, 4356–4367 (2005).

20. A. Nivina, J. A. Escudero, C. Vit, D. Mazel, C. Loot, Efficiency of integron cassette insertion in correct orientation is ensured by the interplay of the three unpaired features of *attC* recombination sites. *Nucleic Acids Res.* **44**, 7792–7803 (2016).

21. C. Loot, M. Ducos-Galand, J. A. Escudero, M. Bouvier, D. Mazel, Replicative resolution of integron cassette insertion. *Nucleic Acids Res.* **40**, 8361–8370 (2012).

22. A. Larouche, P. H. Roy, Analysis by mutagenesis of a chromosomal integron integrase from *Shewanella amazonensis* SB2BT. *J. Bacteriol.* **191**, 1933–1940 (2009).

23. G. Demarre, C. Frumerie, D. N. Gopaul, D. Mazel, Identification of key structural determinants of the IntI1 integron integrase that influence *attC*×*attI1* recombination efficiency. *Nucleic Acids Res.* **35**, 6475–6489 (2007).

24. A. Nivina, M. S. Grieb, C. Loot, D. Bikard, J. Cury, L. Shehata, J. Bernardes, D. Mazel, Structure-specific DNA recombination sites: Design, validation, and machine learning–based refinement. *Sci. Adv.* **6**, eaay2922 (2020).

25. M. S. Grieb, A. Nivina, B. L. Cheeseman, A. Hartmann, D. Mazel, M. Schlierf, Dynamic stepwise opening of integron *attC* DNA hairpins by SSB prevents toxicity and ensures functionality. *Nucleic Acids Res.* **45**, 10555–10563 (2017).

26. C. Loot, V. Parissi, J. A. Escudero, J. Amarir-Bouhram, D. Bikard, D. Mazel, The integron integrase efficiently prevents the melting effect of Escherichia coli single-stranded DNA-binding protein on folded *attC* sites. *J. Bacteriol.* **196**, 762–771 (2014).

27. C. Bustamante, S. Yan, The development of single molecule force spectroscopy: From polymer biophysics to molecular machines. *Q. Rev. Biophys.* **55**, e9 (2022).

28. A. G. T. Pyo, M. T. Woodside, Memory effects in single-molecule force spectroscopy measurements of biomolecular folding. *Phys. Chem. Chem. Phys.* **21**, 24527–24534 (2019).

29. A. Gravel, N. Messier, P. H. Roy, Point mutations in the integron integrase IntI1 that affect recombination and/or substrate recognition. *J. Bacteriol.* **180**, 5437–5442 (1998).

30. C. Johansson, M. Kamali-Moghaddam, L. Sundström, Integron integrase binds to bulged hairpin DNA. *Nucleic Acids Res.* **32**, 4033–4043 (2004).

31. A. Larouche, P. H. Roy, Effect of *attC* structure on cassette excision by integron integrases. *Mob. DNA* **2**, 3 (2011).

32. D. Freedman, R. Pisani, R. Purves, *Statistics: Fourth International Student Edition* (W. W. Norton & Company, 2007).

33. C. Loot, G. A. Millot, E. Richard, E. Littner, C. Vit, F. Lemoine, B. Néron, J. Cury, B. Darracq, T. Niault, D. Lapaillerie, V. Parissi, E. P. C. Rocha, D. Mazel, Integron cassettes integrate into bacterial genomes via widespread non-classical *attG* sites. *Nat. Microbiol.* **9**, 228–240 (2024).

34. K. Jain, E. A. Wood, Z. J. Romero, M. M. Cox, RecA-independent recombination: Dependence on the *Escherichia coli* RarA protein. *Mol. Microbiol.* **115**, 1122–1137 (2021).

35. M. Schlierf, H. Li, J. M. Fernandez, The unfolding kinetics of ubiquitin captured with single-molecule force-clamp techniques. *Proc. Natl. Acad. Sci. U.S.A.* **101**, 7299–7304 (2004).

36. M. D. Wang, M. J. Schnitzer, H. Yin, R. Landick, J. Gelles, S. M. Block, Force and velocity measured for single molecules of rna polymerase. *Science* **282**, 902–907 (1998).

37. J. G. Yodh, M. Schlierf, T. Ha, Insight into helicase mechanism and function revealed through single-molecule approaches. *Q. Rev. Biophys.* **43**, 185–217 (2010).

38. A. Goel, M. D. Frank-Kamenetskii, T. Ellenberger, D. Herschbach, Tuning DNA "strings": Modulating the rate of DNA replication with mechanical tension. *Proc. Natl. Acad. Sci. U.S.A.* **98**, 8485–8489 (2001).

39. P. Thomen, P. J. Lopez, F. Heslot, Unravelling the mechanism of RNA-polymerase forward motion by using mechanical force. *Phys. Rev. Lett.* **94**, 128102 (2005).

40. F. de la Cruz, L. S. Frost, R. J. Meyer, E. L. Zechner, Conjugative DNA metabolism in Gram-negative bacteria. *FEMS Microbiol. Rev.* **34**, 18–40 (2010).

41. T. Q. Trinh, R. R. Sinden, Preferential DNA secondary structure mutagenesis in the lagging strand of replication in *E. coli*. *Nature* **352**, 544–547 (1991).

42. N. G. Bush, K. Evans-Roberts, A. Maxwell, DNA topoisomerases. *EcoSal Plus* **6**, 10.1128/ecosalplus.ESP-0010-2014 (2015).

43. J. K. Lodge, T. Kazic, D. E. Berg, Formation of supercoiling domains in plasmid pBR322. *J. Bacteriol.* **171**, 2181–2187 (1989).

44. Y. Chen, U. Narendra, L. E. Iype, M. M. Cox, P. A. Rice, Crystal structure of a Flp recombinase-Holliday junction complex: Assembly of an active oligomer by helix swapping. *Mol. Cell* **6**, 885–897 (2000).

45. E. Ennifar, J. E. W. Meyer, F. Buchholz, A. F. Stewart, D. Suck, Crystal structure of a wild-type Cre recombinase–*loxP* synapse reveals a novel spacer conformation suggesting an alternative mechanism for DNA cleavage activation. *Nucleic Acids Res.* **31**, 5449–5460 (2003).

46. G. Meinke, J. Karpinski, F. Buchholz, A. Bohm, Crystal structure of an engineered, HIV-specific recombinase for removal of integrated proviral DNA. *Nucleic Acids Res.* **45**, 9726–9740 (2017).

47. A. Bebel, E. Karaca, B. Kumar, W. M. Stark, O. Barabas, Structural snapshots of Xer recombination reveal activation by synaptic complex remodeling and DNA bending. *eLife* **5**, e19706 (2016).

48. M. Zuker, Mfold web server for nucleic acid folding and hybridization prediction. *Nucleic Acids Res.* **31**, 3406–3415 (2003).

49. M. J. Avellaneda, E. J. Koers, D. P. Minde, V. Sunderlikova, S. J. Tans, Simultaneous sensing and imaging of individual biomolecular complexes enabled by modular DNA–protein coupling. *Commun. Chem.* **3**, 20 (2020).

50. M. Swoboda, J. Henig, H.-M. Cheng, D. Brugger, D. Haltrich, N. Plumeré, M. Schlierf, Enzymatic oxygen scavenging for photostability without pH drop in single-molecule experiments. *ACS Nano* **6**, 6364–6369 (2012).

51. M. D. Wang, H. Yin, R. Landick, J. Gelles, S. M. Block, Stretching DNA with optical tweezers. *Biophys. J.* **72**, 1335–1346 (1997).

52. J. Schindelin, I. Arganda-Carreras, E. Frise, V. Kaynig, M. Longair, T. Pietzsch, S. Preibisch, C. Rueden, S. Saalfeld, B. Schmid, J.-Y. Tinevez, D. J. White, V. Hartenstein, K. Eliceiri, P. Tomancak, A. Cardona, Fiji: An open-source platform for biological-image analysis. *Nat. Methods* **9**, 676–682 (2012).

53. A. Gravel, B. Fournier, P. H. Roy, DNA complexes obtained with the integron integrase IntI1 at the *attI1* site. *Nucleic Acids Res.* **26**, 4347–4355 (1998).

54. S. R. Partridge, G. D. Recchia, C. Scaramuzzi, C. M. Collis, H. W. Stokes, R. M. Hall, Definition of the attI1 site of class 1 integrons. *Microbiology* **146**, 2855–2864 (2000).

55. C. Jarzynski, Nonequilibrium equality for free energy differences. *Phys. Rev. Lett.* **78**, 2690–2693 (1997).

56. L. Schrödinger, W. DeLano, PyMOL (2020); http://pymol.org/pymol.
